# Sensitivity, uncertainty and identifiability analyses to define a dengue transmission model with real data of an endemic municipality of Colombia

**DOI:** 10.1371/journal.pone.0229668

**Published:** 2020-03-11

**Authors:** Diana Paola Lizarralde-Bejarano, Daniel Rojas-Díaz, Sair Arboleda-Sánchez, María Eugenia Puerta-Yepes

**Affiliations:** 1 Departamento de Ciencias Matemáticas, Universidad EAFIT, Medellín, Antioquia, Colombia; 2 Departamento de Ciencias Biológicas, Universidad EAFIT, Medellín, Antioquia, Colombia; 3 Grupo de Biología y Control de Enfermedades Infecciosas-BCEI, Universidad de Antioquia, Medellín, Antioquia, Colombia; Syracuse University, UNITED STATES

## Abstract

Dengue disease is a major problem for public health surveillance entities in tropical and subtropical regions having a significant impact not only epidemiological but social and economical. There are many factors involved in the dengue transmission process. We can evaluate the importance of these factors through the formulation of mathematical models. However, the majority of the models presented in the literature tend to be overparameterized, with considerable uncertainty levels and excessively complex formulations. We aim to evaluate the structure, complexity, trustworthiness, and suitability of three models, for the transmission of dengue disease, through different strategies. To achieve this goal, we perform structural and practical identifiability, sensitivity and uncertainty analyses to these models. The results showed that the simplest model was the most appropriate and reliable when the only available information to fit them is the cumulative number of reported dengue cases in an endemic municipality of Colombia.

## Introduction

Dengue is present in tropical and subtropical climates throughout the planet, especially in urban and semi-urban areas [1]. There are four distinct serotypes of the virus that cause dengue (DEN-1, DEN-2, DEN-3 and DEN-4). Symptoms appear between three and fourteen days (average four to seven days) after an infective bite [2]. *Aedes aegypti* is the principal vector for the transmission of Dengue virus in America. The transmission cycle begins when a susceptible female mosquito bites an infectious human. After this, the infected mosquito can transmit the pathogen throughout its life. The symptomatic and asymptomatic humans are the main carriers and multipliers of the virus [1].

Dengue disease has become a major problem for public health surveillance entities having a significant impact not only epidemiological but social and economical [3, 4]. In the last few decades, there has been a surge of interest in the study of the transmission of infectious diseases, as dengue disease, by the formulation of mathematical models based on Ordinary Differential Equations systems (ODEs). This approach provides us with a key instrument to understand, to explain and to determine when an outbreak of the disease occurs and also to be able to design control strategies [5]. For this reason, extra care must be taken to design and to analyze epidemiological models. Although extensive research has been carried out on the formulation of different models of transmission of infectious diseases, few attempts have been made to evaluate the reliability of the results obtained from these models in real scenarios, and the possibility of working with simpler models that answer the same questions but do not require as much information.

Additionally, complex mathematical models can be difficult to fit to data, mainly because they need to process detailed experimental information that is often not available (e.g., weekly reports of mosquitoes being infected). By model complexity, we mean the number of state variables together with the number of parameters. Understanding the model complexity and the nature of its parameters is of vital importance since applying this knowledge to real scenarios can help to develop strategies to assess and select the most appropriate model.

For the transmission of dengue disease, several mathematical models have previously been developed, including considerations such as a variable human population [6], effects of vector control on dengue transmission [7], and the existence of multiple serotypes [8]; a more detailed review is provided in [9, 10]. However, the lack of articulation of these mathematical models with the estimation of their parameters from real data has not allowed for this implementation as a useful tool in the prevention and control of this disease.

This study was designed to analyze and to determine if the parameters estimated in the calibration process of the model from data, are reliable. For this, we performed a combined study of uncertainty, sensitivity, and identifiability (structural and practical) of model parameters. The sensitivity and uncertainty analysis will allow us to identify which parameters are the most relevant for the model output, while the structural and practical identifiability analysis will help us to determine if it is possible to estimate the model parameters uniquely from the available information in two scenarios, with noise-free data, and with noisy data. Moreover, we intend that such models explain the phenomenon under study without losing the biological meaning of the model parameters. From the results of these analyses, we can have arguments to choose a model instead of another.

To achieve this goal we introduced three ODE models for dengue transmission which consider different developmental features of mosquito population. For our case study, given that laboratory data is available, it is possible to define initial ranges for model parameters and initial conditions of each model. Additionally, we considered the cumulative number of reported dengue cases of an endemic municipality of Colombia. After that, we performed sensitivity and uncertainty analysis, and structural and practical identifiability analysis, for each model. Moreover, we calculated and analyzed the basic reproductive number, *R*_0_, which is one of the most important threshold values in mathematical epidemiology [11].

In the case of the structural identifiability analysis, we are able to know if it is possible, under certain conditions (noise-free data and error-free model), to estimate the parameters in a unique way (locally and globally) [12]. This kind of analysis does not require any experimental data. There are several approaches to perform it [13] like direct methods, implicit function based approaches, and Taylor’s generating series. For a deeper discussion, comparison, and details of structural identifiability analysis, we refer the reader to [14]. On the computational approach, as far as we know, we count with various software tools designed to perform structural identifiability analysis of non-linear models: DAISY [15], GenSSI [16], the Identifiability Analysis package in MATHEMATICA [17], COMBOS [18], and STRIKE-GOLDD [19]. However, we could not find a single tool that works for all mathematical models. The selection of a tool to work with depends on its algorithms, as they are designed to exploit different features of the models (like their number of variables, their number of parameters or their being of algebraic type).

On the other hand, practical identifiability analysis aims to help us determine whether the parameters can be uniquely estimated in the presence or non presence of noisy data. The noisy data generation is part of this analysis, from which we determine some level of error (*σ*_0_) to work with. Then, we use this noise to assess the performance of the proposed model. Performance comparison for parameters can be carried out when an indicator, as the average relative estimation error (ARE), determine whether the parameters are identifiable for different levels of noise in the data. Monte Carlo simulations and the calculation of correlation matrices are methodologies used to perform this analysis. For details about these methodologies we refer the reader to Section Practical identifiability analysis.

Another aspect to take into account for the parameter estimation problem is determining when small changes in the value of some parameters have a significant impact on the model output. To address this problem, it is necessary to perform the sensitivity and uncertainty analyses [20]. According to [21], the main goal of sensitivity analysis (SA) is the quantification of the contribution of each parameter to the model output, taking into account the possible values within the parameter range as well as the interactions with other parameters, i.e., the parameter relevance for the model. On the other hand, the uncertainty analysis (UA) attempts to provide a graphical insight about the propagation of uncertainty from the parameters to the model output. There are several approaches for SA, see for instance [22–24]. However, we focus on the variance-based approach since it has proved to be versatile and effective for the relevance ranking of parameters and it is widely implemented in the literature [21, 24]. The variance-based SA is the equivalent of analyzing the model output variance through an experimental design, though the effect of parameters is not estimated over levels but the whole distribution of each parameter respectively, which can be achieved through Monte Carlo simulation approach [24]. However, an informative SA usually requires a large number of model simulations, which constitutes a limitation of the technique.

The paper is organized as follows. First, we introduce three different models for the transmission of dengue, and calculate the basic reproductive number for each model. Then, we present the details of the methodologies to perform the sensitivity, uncertainty, and structural and practical identifiability analyses. After that, we present the results obtained for each model. Finally, we draw our main conclusions and discuss future work. We have added all the MATLAB code used in this study to https://github.com/drojasd/identifiabilityPaperPlos.

## Materials and methods

### Mathematical model

In this section, we introduce three epidemiological models for the transmission of dengue disease. For all these models we consider two populations: mosquito population and human population. We use *M* to denote the size of mosquito population, which can vary over time, and *H* to denote the size of human population, which is considered to remain constant (birth and death rate equal to *μ*_*h*_) over the studied time period (one year). The differences among the models presented in this work are the number of state variables, biological considerations about mosquito populations and parameters. We started with a model based on the work of [25] that considers all stages of development of the vector (eggs *E*, larvae *L*, pupae *P*, and adult phase *M*), followed by a model based on the work of [26] that considers just one aquatic phase *A* (larvae and pupae) and adult phase *M*. For the last model, we went further with the idea of reducing state variables leaving only the adult population of mosquito *M*. For all these models, only female mosquitoes appear in the adult phase. Moreover, this population was divided into three sub-populations, representing susceptible *M*_*s*_, exposed *M*_*e*_, and infectious *M*_*i*_ mosquitoes. Analogously, human population in these models are of four kinds: susceptible *H*_*s*_, exposed *H*_*e*_, infectious *H*_*i*_, and recovered *H*_*r*_.

In the first model (1), the development of mosquito begins with the number of eggs *E* at time *t*, which increases with the per capita oviposition rate $\delta (1-\frac{E}{C})$, where *δ* is the intrinsic oviposition rate per capita, and *C* is the carrying capacity of the environment. The number of eggs decreases according to the transition rate from eggs to larvae *γ*_*e*_ and the eggs’ mortality rate *μ*_*e*_. The number of larvae *L* at time *t* increases with the transition rate from eggs to larvae *γ*_*e*_ and decreases with the transition rate from larvae to pupae *γ*_*l*_ and larvae mortality rate *μ*_*l*_. Likewise, the number of pupae *P* at time *t* increases with the transition rate from larvae to pupae *γ*_*l*_, and decreases with the transition rate from pupae to adults *γ*_*p*_ and the pupae mortality rate *μ*_*p*_. In this manner, the population of adult mosquitoes, including females and males, increases at rate *γ*_*p*_. Because Dengue transmission only involves female mosquito, we included the parameter *f*, which represents the fraction of female mosquitoes produced during hatching of all eggs. Thus, the population of susceptible females *M*_*s*_ increases at rate *fγ*_*p*_, because we removed the number of males *γ*_*p*_(1 − *f*)*P* that completed the development cycle.

In the second model (2), for the mosquito population, we consider larval and pupal stages collectively as the aquatic phase *A*, which increases with the effective per capita oviposition rate $\rho (1-\frac{A}{C})$, with *ρ* = *kδ*, where *δ* is the intrinsic oviposition rate per capita, and *k* is the fraction of eggs hatching to larvae. The aquatic phase decreases according to the transition rate from the aquatic phase to the adult phase *γ*_*m*_ and the mortality rate in the aquatic phase *μ*_*a*_. Similarly to the model (1) the population of susceptible females *M*_*s*_ increases at rate *fγ*_*m*_. Finally, for the third model (3) we only considered the adult population of female mosquitoes. In this model we assume a constant recruitment rate Λ, independent of the actual number of adult mosquitoes. This assumption seems reasonable, since only a fraction of a large reservoir of eggs and larvae matures to females, and this process does not depend directly on the size of the female mosquito population [27].

For these models, dengue transmission begins when a susceptible *Ae. aegypti* female feeds on the blood of an infectious human, thereby becoming an exposed mosquito with a transmission rate $\beta_{m}\;\frac{H_{i}}{H}$, that depends on (a) the transmission coefficient, $\beta_{m}=b\beta_{m}^{\prime }$, where *b* represents the mosquitoes’ biting rate (which is the average number of bites per time unit), and $\beta_{m}^{\prime }$ is the probability of a mosquito becomes infected after biting a human with Dengue and (b) the proportion of infectious humans, *H*_*i*_/*H*. The exposed mosquito becomes infectious when the extrinsic incubation period is completed, which occurs at a rate *θ*_*m*_, where 1/*θ*_*m*_ is the average duration of the extrinsic incubation period. Analogously, susceptible humans become exposed humans at a rate $\beta_{h}\;\frac{M_{i}}{M}$, where the transmission coefficient is given by $\beta_{h}=b\beta_{h}^{\prime }$ where *β*_*h*_ is the probability that a person becomes infected after being bitten by an infectious mosquito with Dengue. We followed the scheme proposed by [25] rather than that proposed by [26] for transmission dynamics since treating the transition rate from susceptible to exposed humans as $\beta_{h}\;\frac{H_{i}}{H}M_{i}$, as in [26] and other papers, makes a dimensional issue arise, i.e., the units of the term are $\frac{mosquitoes}{time}$, while the units of their respective state equation are $\frac{humans}{time}$. For a better insight into the implications of dimensional analysis, we refer the reader to [28]. When the intrinsic incubation period is completed, the exposed human becomes infectious, which occurs at a rate *θ*_*h*_, where 1/*θ*_*h*_ is the average duration of the intrinsic incubation period. Finally, the infectious humans recover at a rate *γ*_*h*_, where 1/*γ*_*h*_ is the average duration of the recovery period [6]. Models based on these assumptions are given by the following systems of ordinary differential equations, where the variable *t* (time) is measured in weeks:

- Model (1)
  $$\begin{array}{c} \begin{array}{cc} \frac{dE}{dt} & =\delta (1-\frac{E}{C})M-\left(\gamma_{e}+\mu_{e}\right)E\\ \frac{dL}{dt} & =\gamma_{e}E-\left(\gamma_{l}+\mu_{l}\right)L\\ \frac{dP}{dt} & =\gamma_{l}L-\left(\gamma_{p}+\mu_{p}\right)P\\ \frac{dM_{s}}{dt} & =f\gamma_{p}P-\beta_{m}\frac{H_{i}}{H}M_{s}-\mu_{m}M_{s}\\ \frac{dM_{e}}{dt} & =\beta_{m}\frac{H_{i}}{H}M_{s}-\left(\theta_{m}+\mu_{m}\right)M_{e}\\ \frac{dM_{i}}{dt} & =\theta_{m}M_{e}-\mu_{m}M_{i}\\ \frac{dH_{s}}{dt} & =\mu_{h}H-\beta_{h}\frac{M_{i}}{M}H_{s}-\mu_{h}H_{s}\\ \frac{dH_{e}}{dt} & =\beta_{h}\frac{M_{i}}{M}H_{s}-\left(\theta_{h}+\mu_{h}\right)H_{e}\\ \frac{dH_{i}}{dt} & =\theta_{h}H_{e}-\left(\gamma_{h}+\mu_{h}\right)H_{i}\\ \frac{dH_{r}}{dt} & =\gamma_{h}H_{i}-\mu_{h}H_{r} \end{array} \end{array}$$ (1)
- Model (2)
  $$\begin{array}{c} \begin{array}{cc} \frac{dA}{dt} & =\rho (1-\frac{A}{C})M-\left(\gamma_{m}+\mu_{a}\right)A\\ \frac{dM_{s}}{dt} & =f\gamma_{m}A-\beta_{m}\frac{H_{i}}{H}M_{s}-\mu_{m}M_{s}\\ \frac{dM_{e}}{dt} & =\beta_{m}\frac{H_{i}}{H}M_{s}-\left(\theta_{m}+\mu_{m}\right)M_{e}\\ \frac{dM_{i}}{dt} & =\theta_{m}M_{e}-\mu_{m}M_{i}\\ \frac{dH_{s}}{dt} & =\mu_{h}H-\beta_{h}\frac{M_{i}}{M}H_{s}-\mu_{h}H_{s}\\ \frac{dH_{e}}{dt} & =\beta_{h}\frac{M_{i}}{M}H_{s}-\left(\theta_{h}+\mu_{h}\right)H_{e}\\ \frac{dH_{i}}{dt} & =\theta_{h}H_{e}-\left(\gamma_{h}+\mu_{h}\right)H_{i}\\ \frac{dH_{r}}{dt} & =\gamma_{h}H_{i}-\mu_{h}H_{r} \end{array} \end{array}$$ (2)
- Model (3)
  $$\begin{array}{c} \begin{array}{cc} \frac{dM_{s}}{dt} & =\Lambda -\beta_{m}\frac{H_{i}}{H}M_{s}-\mu_{m}M_{s}\\ \frac{dM_{e}}{dt} & =\beta_{m}\frac{H_{i}}{H}M_{s}-\left(\theta_{m}+\mu_{m}\right)M_{e}\\ \frac{dM_{i}}{dt} & =\theta_{m}M_{e}-\mu_{m}M_{i}\\ \frac{dH_{s}}{dt} & =\mu_{h}H-\beta_{h}\frac{M_{i}}{M}H_{s}-\mu_{h}H_{s}\\ \frac{dH_{e}}{dt} & =\beta_{h}\frac{M_{i}}{M}H_{s}-\left(\theta_{h}+\mu_{h}\right)H_{e}\\ \frac{dH_{i}}{dt} & =\theta_{h}H_{e}-\left(\gamma_{h}+\mu_{h}\right)H_{i}\\ \frac{dH_{r}}{dt} & =\gamma_{h}H_{i}-\mu_{h}H_{r} \end{array} \end{array}$$ (3)
  where *M*(*t*) = *M*_*s*_(*t*) + *M*_*e*_(*t*) + *M*_*i*_(*t*) and *H* = *H*_*s*_(*t*) + *H*_*e*_(*t*) + *H*_*i*_(*t*) + *H*_*r*_(*t*). In S1 Fig we include a flowgraph for each model where all transitions described above are shown.

### Basic reproductive number

The basic reproductive number *R*_0_ is one of the most important quantities in the study of epidemics. This quantity is defined as the expected number of new cases of an infection caused by a typical infectious individual in a population consisting of susceptible only [29].

For the computation of *R*_0_, we follow the strategy presented in [30]. We outline the strategy to calculate *R*_0_ in the following steps.

1. Identify the *ODE* system that describes the production of new infections and changes in state among infected individuals. We will refer to the set of such equations as the infected subsystem.
2. Linearize the infected subsystem of nonlinear ODEs about the infection-free steady state. This linear system can be described by a matrix. In this paper we refer to this matrix as the Jacobian matrix of the infected system and we denote it by *J*.
3. Rewrite the matrix *J* as *T* + Σ, where *T* is the *transmission part*, describing the production of new infections, and Σ is the *transition part*, describing changes in state (including removal by death or the acquisition of immunity).
4. Finally, we compute the dominant eigenvalue, or more precisely the spectral radius *ρ*, of the matrix **K** = −*T*Σ^−1^. The matrix **K** is called the *next-generation matrix* (NGM) and *R*_0_ the dominant eigenvalue of this matrix [31].

### Data and parameters values

In this study, we consider the cumulative number of reported dengue cases of Bello (Antioquia-Colombia) from epidemiological week 49 of 2009 (with 8 reported cases) to epidemiological week 7 of 2011 (with 3 reported cases). The number of total cases reported during this period was 1880, which according to local surveillance entities, is classified as an outbreak for Bello municipality.

To define the entomological parameters range for each model we consider the results of life tables obtained from experimental assays performed with mosquitoes population of Bello in the BCEI (Grupo de Biología y Control de Enfermedades Infecciosas de la Universidad de Antioquia) in 2017. For more detailed information about the experimental protocol, we refer to [32] and [33]. Range of values for the intrinsic incubation period, extrinsic incubation period, and recovery rate were taken from the literature [2]. The parameter ranges are summarized in Table 1.

**Table 1:** Parameters used in the simulations of models (1)–(3), their biological descriptions, and their value ranges.

Model	Parameters	Meaning	Values per day	Values per week
Model (1)	*δ*	Per capita oviposition rate	[20, 60]	[20, 240]
	*γ*_*e*_	Transition rate from eggs to larvae	[0.2, 0.5]	[1.4, 3.5]
	*μ*_*e*_	Mortality rate in eggs phase	[0, 0.015]	[0, 0.11]
	*γ*_*l*_	Transition rate from larvae to pupae	[0.2, 0.2]	[1.4, 1.4]
	*μ*_*l*_	Mortality rate in the larvae phase	[0.004, 0.03]	[0.028, 0.18]
	*γ*_*p*_	Transition rate from pupae to the adult phase	[0.33, 0.5]	[2.33, 3.5]
	*μ*_*p*_	Mortality rate in pupae phase	[0.008, 0.083]	[0.054, 0.58]
Model (2)	*ρ*	Effective per capita oviposition rate	[19, 60]	[19, 240]
	*γ*_*m*_	Transition rate from the aquatic phase to the adult phase	[0.125, 0.143]	[0.875, 1]
	*μ*_*a*_	Mortality rate in the aquatic phase	[0.0025, 0.023]	[0.017, 0.16]
Model (3)	Λ	Recruitment rate	[278, 6110]	[1881, 42694]
Model (1) and (2)	*f*	Fraction of female mosquitoes hatched from all eggs	[0.35, 0.45]	[0.35, 0.45]
	*C*	Carrying capacity of the environment	[6400, 95000]	[6400, 95000]
Model (1)-(3)	*μ*_*m*_	Mortality rate in the adult phase	[0.019, 0.023]	[0.16, 0.20]
	*μ*_*h*_	Birth and death rate of the human population	0.00006	0.0004
	*β*_*h*_	Transmission rate from mosquito to human	[0, 4]	[0, 4]
	*β*_*m*_	Transmission rate from human to mosquito	[0, 4]	[0, 4]
	*θ*_*m*_	Transition rate from exposed to infectious mosquitoes	[0.08, 0.13]	[0.58, 0.88]
	*θ*_*h*_	Transition rate from exposed to infectious humans	[0.1, 0.25]	[0.7, 1.75]
	*γ*_*h*_	Recovery rate	[0.07, 0.25]	[0.5, 1.75]

Finally, to define the ranges of initial conditions for each model we considered for the aquatic phase of the vector, mosquitoes (susceptible, exposed and infectious), and human (susceptible, exposed, infectious and recovered) the same ranges as in [33]. For the model (1) we defined the initial condition for eggs, larvae and pupae between zero and the maximal value of the carrying capacity, 95000. Initial conditions for models (1)–(3) are summarized in the Table 2.

**Table 2:** Initial conditions used in the simulations of models (1)–(3), their descriptions, and their value ranges.

Model	Initial condition	Meaning	Range
Model (1)	*E*_0_	Eggs	[0, 95000]
	*L*_0_	Larvae	[0, 95000]
	*P*_0_	Pupae	[0, 95000]
Model (2)	*A*_0_	Aquatic phase	[5755, 17265]
Model (1)-(3)	$M_{s_{0}}$	Susceptible mosquitoes	[0, 1200000]
	$M_{e_{0}}$	Exposed mosquitoes	[0, 100]
	$M_{i_{0}}$	Infectious mosquitoes	[0, 100]
	$H_{s_{0}}$	Susceptible humans	[244402, 321734]
	$H_{e_{0}}$	Exposed humans	[18, 72]
	$H_{i_{0}}$	Infectious humans	[6, 24]
	$H_{r_{0}}$	Recovered humans	[81405, 158809]

### Parameter estimation

To fit the transmission dengue models to data we first implemented them into MATLAB environment using the Symbolic Math Toolbox [34] and the GSUA-CSB Toolbox [35]. Then, we solved the parameter estimation problem in the least-squares sense, which is given by the optimization problem:

$$\begin{array}{c} \hat{\theta }=\min_{\theta }\sum_{i=1}^{n}{\left(y_{i},h\left(x\left(t_{i}\right),\theta \right)\right)}^{2} \end{array}$$ (4)

where, *y*_*i*_ with *i* = 1, …, *n* are the observations of cumulative reported dengue cases at time *t*_*i*_, and *h*(**x**(*t*_*i*_), ***θ***) are the output of the cumulative infected humans for each model for the vector of parameters ***θ***.

For the estimation process itself, we used the gsua_pe function of the GSUA-CSB Toolbox with the following parameterization: ODE45 as the MATLAB ODEs solver (this solver applies the so-called *Dormand-Prince* method with a time-step variable of which we get an accuracy improvement during computation [36]). To solve the least-squares problem, we used the optimization algorithm implementation lsqcurvefit with mean squared error (MSE) as the cost function and the trust region reflective as the optimization method, setting the maximum number of model evaluations up to 4000 to ensure convergence (based on previous models assessments).

On the other hand, to avoid local minima, we implemented a hybrid methodology based on advice from [37]. This approach suggests preliminary exploration of the search space through Monte Carlo simulations to discard non-informative regions before the estimation process. Therefore, we did not search for space regions but perform multiple optimizations starting from random parameter values ***θ***. We tried to retrieve as much information as possible from the whole search space, by sampling the random starting parameter values with a Latin-hypercube design. We refer the reader to [38] for further reading about this sample scheme and its advantages. In summary, as local minima avoiding strategy, we performed 1000 parameter estimation tasks for each model and then, we sorted the estimations to keep those that did not overcome the threshold given by (best cost function) × 1.01. In this way, we assumed that there were no remarkable differences among those estimations under the threshold, i.e., those estimations belong to the global minimum.

The aforementioned hybrid methodology allowed us to test, to some extent, the practical identifiability of the models, as we explain in Section Practical identifiability analysis. It is clear that getting reliable analysis, including parameter estimation, requires a large number of samples (starting parameter values in this case). However, computing cost issues arise when attempting to solve these optimization problems as with models (1) and (2). Bearing in mind that for each optimization process alone it is required up to 4000 model simulations to meet the optimization criteria, we performed a small test in order to estimate the average required time per simulation over AMD A12-9700p Notebook, 2.4GHz and 12Gb RAM. Under these computer settings, the average simulation time for each model was about 0.05 sec for (3), 1.59 sec for (2) and 6.91 sec for (1). Consequently, for model (1), more than seven hours would be required per estimation. Of course, the simulation times can be less critical when high-performance computing (HPC) is used. For this reason we implemented HPC for our simulations through Apolo Scientific Computing Center, using 96 nodes and 384Gb of RAM; in this way, we were able to perform all the estimation tasks that we required for the present work (24000).

### Uncertainty and sensitivity analyses

Uncertainty analysis (UA) and sensitivity analysis (SA) are tools to assess and to quantify the uncertainty spread from the input factors (parameters and initial states) to the model output, taking into account the effect of the interactions among those factors [24, 39]. In this work, we treated UA as a graphical assessment of uncertainty propagation based on simple Monte Carlo simulation, i.e., random sampling of factors values from previously defined ranges using a Latin hypercube design; we refer the reader to [38] for further information about this technique. This also allows us to state a range for scalar model output in cases where it was considered relevant. On the other hand, we chose a global approach for SA instead of the local one, because the first attempts to quantify the uncertainty contribution of the model factors in their entire distribution range (space of factors) while the second is only informative for a single point of the space of factors [20].

For this work, we chose a global variance-related SA method proposed in [40] and implemented in the function gsua_sa from GSUA-CSB toolbox [35], which is especially useful for time-response model outputs. Both variance-based and variance-related SA are usually improvements of Sobol [41, 42]. The so-called Sobol method is based on decomposing the variance of model output into terms of increasing dimensionality (HDMR). Then, it is possible to find the contribution to output variance of each factor and its interactions. See a detailed framework for SA in S1 File. Also, when performing SA, it is common to calculate two normalized-index sets: the first-order sensitivity indices (*S*_*i*_) that quantify the contribution of each factor to model output; and the total-order sensitivity indices ($S_{t_{i}}$) that quantify the contribution of each factor alone and all of its interactions. It follows that $S_{i}\leq S_{t_{i}}$, and both terms tend to be equal as the aforementioned interactions become negligible (see S1 File). When ∑_*i*_ *S*_*i*_ tends to one, we shall say no strong interactions occur in the model, and a local approach must suffice to give a good picture of the model behavior. Besides, we shall say, following [20] that a model is *relevant* when its relevance measure, Ψ in (5), tends to one.

$$\begin{array}{c} \Psi =\frac{\text{number}\;\text{of}\;\text{parameters}\;\text{whose}\;S_{t_{i}}\neq 0}{\text{total}\;\text{number}\;\text{of}\;\text{parameters}\;\text{of}\;\text{the}\;\text{model}} \end{array}$$ (5)

Variance-based SA requires a large number of samples of the space of parameters (which is defined by the range of the parameters): the greater the sample size, the greater the reliability of the SA. Hence, it is necessary to establish a reliability measure. The sensitivity index estimator we chose from [24] allows to get negative first-order sensitivity indices (*S*_*i*_). However, by definition, the sensitivity indices can not be negative. Those negative values for *S*_*i*_ could be reached when trying to estimate indices for the first-order non-relevant parameters. Then, we can define a reliability indicator in the form (∑_*i*∈***θ***_ *S*_*i*_/∑_*i*∈***θ***_|*S*_*i*_|) × (100%). In this way, as the reliability estimator tends to one, we can say that the sample size is large enough to estimate reliable sensitivity indices. We consider that the reliability indicator we proposed is a good approach indeed, since epidemiological models are usually overparameterized, and then it is likely for several parameters to have their respective *S*_*i*_ close to zero.

We were particularly interested in SA as a criterion for model validation since it has been reported a link between identifiability and SA theory, see [43]. Briefly, if a factor is non-influential for model output, then it could take any value within its range during the estimation process; hence, by definition, the factor is unidentifiable in practice. A factor can be influential and unidentifiable, but this corresponds to another kind of identifiability that we address later. Summarizing, we applied global SA to achieve three different goals. First, to assess the relevance of global approaches for future studies of the models we expose here. Second, as an indicator of unidentifiable and non-informative factors. Third, as an indicator of those factors that carry the most of model information and hence determine its behavior.

### Structural identifiability analysis

*“In many sciences, it is possible to conduct experiments to obtain information and test hypotheses. However, experiments with the spread of infectious diseases in human populations are often impossible, unethical, or expensive.”* [5]. Therefore, we can not measure all parameters of the models through experimental assays. However, indirect approaches such as parameter estimation methods can help us to determine these parameters values using the available information. To know if it is possible to estimate unique values to model unknown parameters from the available observables (assuming noise-free data and error-free model) we have to performance a study of structural identifiability analysis. This analysis does not require experimental data. For that reason this analysis is called *a prior analysis* and can be used to help to design experimental assays and determine which information should be collected. To set up this problem, we considered the models (1)–(3) in the following form:

$$\begin{array}{c} \dot{x}\left(t\right)=f\left(t,x\left(t\right),\theta \right),\;\;\;x\left(0\right)=x_{0} \end{array}$$ (6)

where ***θ*** denotes the parameters of the system, **x**(*t*) is the vector of state variables, and **x**_0_ is the initial values. The cumulative number of dengue cases are given by the output function **h**(**x**(*t*), ***θ***). To establish what it means for a system to be *structural identifiable* we introduced the following definitions taken from [13].

**Definition 1**. A system structure (6) is said to be *globally identifiable* if for any two parameter vectors ***θ***_1_ and ***θ***_2_ in the parameter space, **h**(**x**(*t*), ***θ***_1_) = **h**(**x**(*t*), ***θ***_2_) holds if and only if ***θ***_1_ = ***θ***_2_.

**Definition 2**. A system structure (6) is said to be *locally identifiable* if for any ***θ*** within an open neighborhood of some point θ* in the parameter space, **h**(**x**(*t*), ***θ***_1_) = **h**(**x**(*t*), ***θ***_2_) holds if and only if ***θ***_1_ = ***θ***_2_.

In this study, we use the *Identifiability Analysis* package in Mathematica software to test for the local identifiability of the epidemiological models of dengue transmission (1)–(3). This implementation is based on a probabilistic numerical method of computing the rank of the identifiability (Jacobian) matrix where the matrix parameters and initial state variables are specialized to random integers. For more detailed information, we refer to [17].

### Practical identifiability analysis

In contrast to structural identifiability analysis, in the practical approach we can consider noise in the experimental data to evaluate the reliability of the parameter estimation [13]. As is stated in [44] we define the practical identifiability problem as follows. Given a dynamical model described by

$$\begin{array}{c} \begin{array}{cc} \dot{x} & =f\left(x\left(t\right),\theta \right)\;x\left(t_{0}\right)=x_{0}\\ y & =h\left(x\left(t\right),\theta \right)+\epsilon \left(t\right)≔\hat{y}\left(t,\theta \right)+\epsilon \left(t\right) \end{array} \end{array}$$ (7)

with state $x\left(t\right)\in R^{n}$, output $y\left(t\right)\in R^{m}$, random measurement noise $∊\left(t\right)\in R^{m}$, and unknown parameter vector $\theta \in R^{p}$, assuming a finite set of *N* input-output measurements are available, form the average weighted square prediction error

$$\begin{array}{c} V_{N}\left(\theta \right)≔\frac{1}{N}\sum_{k=1}^{N}{\left[y\left(t_{k}\right)-\hat{y}\left(t_{k},\theta \right)\right]}^{T}Q_{k}\left[y\left(t_{k}\right)-\hat{y}\left(t_{k},\theta \right)\right] \end{array}$$ (8)

where *Q*_*k*_ are positive semidefinite weights. One says that the system (or the parameter ***θ***) is practically identifiable if *V*_*N*_(***θ***) has a unique minimum. If the error terms *ϵ*(*t*) are assumed to be Gaussian the function *V*_*N*_(***θ***) is essentially the likelihood function of the experiment.

In this study, we applied three different approaches to determine if a parameter is practically identifiable or not. First, we perform *Monte Carlo simulations* which have been widely used for practical identifiability of ODEs [13]. In general, a Monte Carlo simulation procedure can be outlined as follows:

1. Determine the nominal parameters values $\hat{\theta }$, which can be obtained by fitting the epidemiological models (1)–(3) to the weakly (cumulative) number of reported dengue cases.
2. Solve the epidemiological models (1)–(3) numerically with the vector of parameters $\hat{\theta }$ (true parameters) to get the output $h(x\left(t\right),\hat{\theta })$ at the discrete data time points *t*_*i*_, with *i* = 1, …*n*.
3. Generate *N* sets of simulated data set with a given measurement error (in this case we assume the error follows a normal distribution with mean 0 and variance *σ*^2^(*t*).
4. Calculate the average relative estimation error (ARE) for each element of ***θ*** as
  $$\begin{array}{c} \text{ARE}\left(\theta^{\left(k\right)}\right)=100\%\times \frac{1}{N}\sum_{i=1}^{N}\frac{|\theta_{0}^{\left(k\right)}-{\hat{\theta }}_{i}^{\left(k\right)}|}{|\theta_{0}^{\left(k\right)}|}, \end{array}$$ (9)
  where $\theta_{0}^{\left(k\right)}$ is the k-th element of the vector ***θ***_0_ and ${\hat{\theta }}_{i}^{\left(k\right)}$ is the k-th element of ${\hat{\theta }}_{i}$.
5. Repeat steps 2 through 4 increasing the level of noise.

As in [12], we said that if the ARE of the parameter is less than the measurement error *σ*_0_, then the parameter is practically identifiable. The ARE can be used to assess whether each of the parameter estimates is acceptable or not [13]. However, there is not a clear way to determine from the ARE value if a parameter is practical identifiable or not. Thus the practical identifiability relies on the underlying problem and judgment of the investigators [13].

Second, we analyze the correlations between parameters. For this, we consider the parameters $\hat{\theta }$ as in the first step in the Monte Carlo simulation. The corresponding correlation matrix, *S*, for these parameters, can be calculated based on the Pearson correlation index, as mentioned in [45], where each component of this matrix, *s*_*ij*_, gives the correlation coefficient between the parameters ${\hat{\theta }}_{i}$ and ${\hat{\theta }}_{j}$. When *s*_*ij*_ is close to 1, we say the parameters ${\hat{\theta }}_{i}$ and ${\hat{\theta }}_{j}$ are *strongly correlated*, which means, these parameters can not be practically identifiable.

And third, we draw a boxplot for the filtered estimations of each model. We think this is a good approach to practical identifiability analysis since we assume that those estimations whose cost function are below a given threshold belong to the same minimum. In this way, a parameter is identifiable to the extent its boxplot tends to be a single point. The higher is the boxplot for a given parameter, the lesser would be its identifiability. Additionally, the presence of unidentifiable parameters in this approach also suggest that the model has multiple global minima.

## Results

### Basic reproductive number

Models (1)–(3) have four infected states: exposed mosquitoes *M*_*e*_, infectious mosquitoes *M*_*i*_, exposed human *H*_*e*_, and infectious human *H*_*i*_. The infected subsystem associated with these models is given by:

$$\begin{array}{c} \begin{array}{cc} \frac{dM_{e}}{dt} & =\beta_{m}\frac{H_{i}}{H}M_{s}-\left(\theta_{m}+\mu_{m}\right)M_{e}\\ \frac{dM_{i}}{dt} & =\theta_{m}M_{e}-\mu_{m}M_{i}\\ \frac{dH_{e}}{dt} & =\beta_{h}\frac{M_{i}}{M}H_{s}-\left(\theta_{h}+\mu_{h}\right)H_{e}\\ \frac{dH_{i}}{dt} & =\theta_{h}H_{e}-\left(\gamma_{h}+\mu_{h}\right)H_{i} \end{array} \end{array}$$ (10)

Moreover, for the model (1) the *infection-free steady state* is given by

$$\begin{array}{c} P_{0}^{1}=\left(E^{*},L^{*},P^{*},M_{s}^{*},M_{e}^{*},M_{i}^{*},H_{s}^{*},H_{e}^{*},H_{i}^{*}\right) \end{array}$$ (11)

where $E^{*}=C(1-\frac{1}{R_{m}})$ with $R_{m}=\frac{f\gamma_{p}}{\mu_{m}}\frac{\gamma_{l}}{\gamma_{p}+\mu_{p}}\frac{\gamma_{e}}{\gamma_{l}+\mu_{l}}\frac{\delta }{\gamma_{e}+\mu_{e}}$, $L^{*}=\frac{\gamma_{e}}{\gamma_{l}+\mu_{l}}E^{*}$, $P^{*}=\frac{\gamma_{l}}{\gamma_{p}+\mu_{p}}L^{*}$, $M_{s}^{*}=\frac{f\gamma_{p}}{\mu_{m}}P^{*}$, $M_{e}^{*}=M_{i}^{*}=0$, $H_{s}^{*}=H$, and $H_{e}^{*}=H_{i}^{*}=0$.

For model (2) the *infection-free steady state* is stated as follows

$$\begin{array}{c} P_{0}^{2}=\left(A^{*},M_{s}^{*},M_{e}^{*},M_{i}^{*},H_{s}^{*},H_{e}^{*},H_{i}^{*}\right)=(A^{*},\frac{f\gamma_{m}}{\mu_{m}}A^{*},0,0,H,0,0) \end{array}$$ (12)

where $A^{*}=C(1-\frac{1}{R_{m}})$ and $R_{m}=\frac{\rho f\gamma_{m}}{\mu_{m}\left(\gamma_{m}+\mu_{a}\right)}$.

Whereas for model (3) the *infection-free steady state* is given by

$$\begin{array}{c} P_{0}^{3}=\left(M_{s}^{*},M_{e}^{*},M_{i}^{*},H_{s}^{*},H_{e}^{*},H_{i}^{*}\right)=(\frac{\Lambda }{\mu_{m}},0,0,H,0,0). \end{array}$$ (13)

Then, the linearization of (10) around the *infection-free steady state* for all three models can be stated as

$$\begin{array}{c} \dot{x}=\left(T+\Sigma \right)x \end{array}$$

where **x** = [*M*_*e*_ *M*_*i*_ *H*_*e*_ *H*_*i*_]^*T*^, $T=(\begin{array}{cccc} 0 & 0 & 0 & \beta_{m}\frac{M_{s}^{*}}{H}\\ 0 & 0 & 0 & 0\\ 0 & \beta_{h}\frac{H_{s}^{*}}{M} & 0 & 0\\ 0 & 0 & 0 & 0 \end{array})$ is the transmission matrix, and $\Sigma =(\begin{array}{cccc} -(\theta_{m}+\mu_{m}) & 0 & 0 & 0\\ \theta_{m} & -\mu_{m} & 0 & 0\\ 0 & 0 & \left(\theta_{h}+\mu_{h}\right) & 0\\ 0 & 0 & \theta_{h} & -(\gamma_{h}+\mu_{h}) \end{array})$ is the transition matrix.

Hence the NGM matrix **K** is four-dimensional and it is

$$\begin{array}{c} \begin{array}{cc} K & =-T\Sigma^{-1}\\ & =(\begin{array}{cccc} 0 & 0 & 0 & \beta_{m}\frac{M_{s}^{*}}{H}\\ 0 & 0 & 0 & 0\\ 0 & \beta_{h}\frac{H_{s}^{*}}{M} & 0 & 0\\ 0 & 0 & 0 & 0 \end{array})\;(\begin{array}{cccc} \frac{1}{\left(\theta_{m}+\mu_{m}\right)} & 0 & 0 & 0\\ \frac{\theta_{m}}{\mu_{m}\left(\theta_{m}+\mu_{m}\right)} & \frac{1}{\mu_{m}} & 0 & 0\\ 0 & 0 & \frac{1}{\left(\theta_{h}+\mu_{h}\right)} & 0\\ 0 & 0 & \frac{\theta_{h}}{\left(\theta_{h}+\mu_{h}\right)\left(\gamma_{h}+\mu_{h}\right)} & \frac{1}{\left(\gamma_{h}+\mu_{h}\right)} \end{array})\\ & =(\begin{array}{cccc} 0 & 0 & \frac{\beta_{m}\theta_{h}}{\left(\theta_{h}+\mu_{h}\right)\left(\gamma_{h}+\mu_{h}\right)}\frac{M_{s}^{*}}{H} & \frac{\beta_{m}}{\left(\gamma_{h}+\mu_{h}\right)}\frac{M_{s}^{*}}{H}\\ 0 & 0 & 0 & 0\\ \frac{\beta_{h}\theta_{m}}{\mu_{m}\left(\theta_{m}+\mu_{m}\right)}\frac{H_{s}^{*}}{M} & \frac{\beta_{h}}{\mu_{m}}\frac{H_{s}^{*}}{M} & 0 & 0\\ 0 & 0 & 0 & 0 \end{array}) \end{array} \end{array}$$

The eigenvalues of **K** are zero of multiplicity 2, and

$$\begin{array}{c} \pm \sqrt{\frac{\beta_{m}\beta_{h}\theta_{m}\theta_{h}}{\mu_{m}\left(\theta_{m}+\mu_{m}\right)\left(\theta_{h}+\mu_{h}\right)\left(\gamma_{h}+\mu_{h}\right)}\frac{H_{s}^{*}M_{s}^{*}}{HM}}. \end{array}$$ (14)

Therefore,

$$\begin{array}{c} R_{0}=\sqrt{\frac{\beta_{m}\beta_{h}\theta_{m}\theta_{h}}{\mu_{m}\left(\theta_{m}+\mu_{m}\right)\left(\theta_{h}+\mu_{h}\right)\left(\gamma_{h}+\mu_{h}\right)}} \end{array}$$ (15)

because in the *infection-free steady state*, $H_{s}^{*}=H$ and $M_{s}^{*}=M$.

### Model fitting and parameter estimation

**Fig 1:**
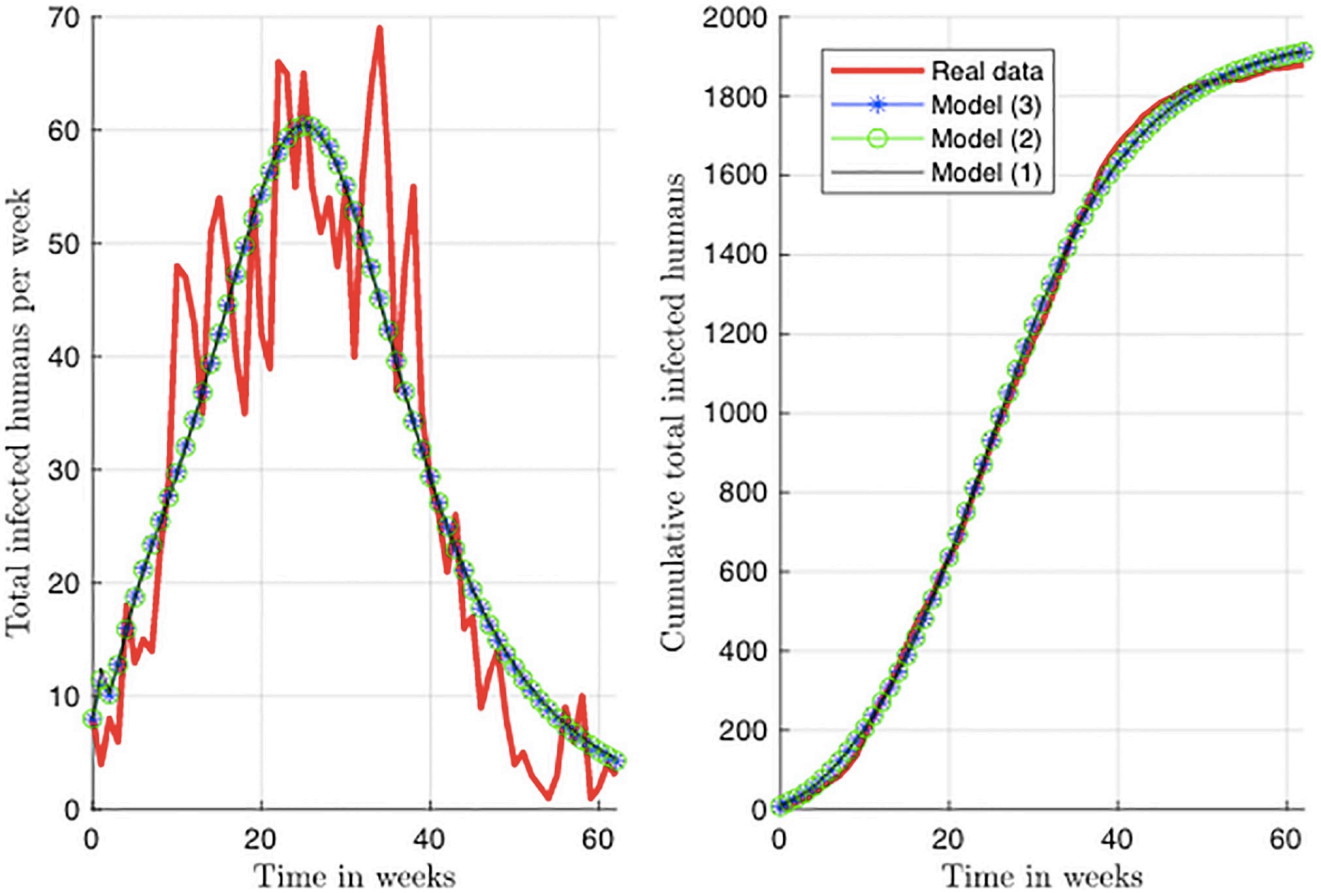
Best fit of models (1)–(3) to reported dengue cases in Bello. The real data corresponds to the number of reported cases for the dengue outbreak occurred in Bello in 2010. We present here two different visualizations to assess the fit of the three models to the data: in the figure to the left we show the fit to the synthetic model state we added ($H_{i_{t}}$), that represents the cumulative number of reported Dengue cases. On the other hand, for the figure in right, we transform the data into cumulative Dengue cases per week, which is a friendly way to visualize the process. We fit the model to the cumulative number of Dengue cases, but we reported the MSE cost function for both of the visualizations as follows: MSE = (value for figure in right- value for figure in left). Cost functions (MSE): model (1) = (75.32-699.49), model (2) = (73.86-658.1), model (3) = (73.22-642.63).

We fitted models (1)–(3) to the cumulative reported dengue cases in Bello municipality, starting from epidemiological week 49 of 2009 (with 8 reported cases) to epidemiological week 7 of 2011 (with 3 reported cases) (Fig 1). For this, we added a new state variable, $H_{i_{t}}=\theta_{h}H_{e}$ for each model that represents the number of cumulative dengue cases without taking into account those recovered. We proceed in this way since the available information is about the new number of reported dengue cases per week and we did not know a priori what the average period of recovery was for this population. Thus, it is not possible to know how many humans were infected for a given time. As stated in the methodology Section Parameter estimation, we performed 1000 parameter estimation tasks for each model starting from different initial points, and then, we filtered the estimations according to the proposed threshold (1% of dissimilarity regards to the best estimation for each model). We kept 136 estimations for model (1) with a standard deviation (std) for MSE cost function of 1.88, 158 estimations for model (2) with a std of 1.92, and 476 estimations for model (3) with a std of 2.12. From now on, when we talk about estimated parameters for any of the models, we will focus on the filtered estimations instead of the whole estimations. As it can be seen in Fig 1, these models captured the overall behavior of reported dengue cases in this municipality. Table 3 shows the best fitted parameter values for each model. Instead of considering the average of the estimations, we include the median for each parameter since this is a more robust estimator [46]. It is worth pointing out that the best fit is near to the median value in almost every parameter in each model.

**Table 3:** Estimated parameters and initial condition for models (1)–(3).

Parameter	Range		Model (1)		Model (2)		Model (3)	
			Estimated	Median	Estimated	Median	Estimated	Median
*E*_0_	0	95000	95000.000	94938.059	-	-	-	-
*L*_0_	0	95000	95000.000	94951.047	-	-	-	-
*P*_0_	0	95000	95000.000	94939.135	-	-	-	-
*A*_0_	5755	17265	-	-	17216.859	16273.478	-	-
$M_{s_{0}}$	0	1200000	1200000.000	1199879.050	1200000.000	1199986.954	1200000.000	1199999.996
$M_{e_{0}}$	0	100	32.938	82.656	31.839	79.411	44.600	75.460
$M_{i_{0}}$	0	100	0.000	0.971	0.000	0.000	0	0
$H_{s_{0}}$	244402	321734	301842.132	290732.912	304026.086	288261.994	318001.356	289123.066
$H_{e_{0}}$	18	72	18.000	18.014	18.000	18.013	18.000	18.000
$H_{r_{0}}$	81405	158809	81405.000	117887.169	81407.485	122431.455	81439.356	83824.309
*δ*	20	240	20.000	20.000	-	-	-	-
*γ*_*e*_	1.4	3.5	1.400	1.409	-	-	-	-
*γ*_*p*_	2.33	3.5	2.330	2.335	-	-	-	-
*μ*_*e*_	0	0.11	0.110	0.110	-	-	-	-
*μ*_*l*_	0.028	0.18	0.180	0.179	-	-	-	-
*μ*_*p*_	0.054	0.58	0.580	0.579	-	-	-	-
*C*	6400	95000	6400.000	6595.133	6400.000	6401.212	-	-
*f*	0.35	0.45	0.350	0.351	0.350	0.350	-	-
*ρ*	19	240	-	-	19.000	19.000	-	-
*γ*_*m*_	0.875	1	-	-	0.875	0.876	-	-
*μ*_*a*_	0.017	0.16	-	-	0.160	0.160	-	-
Λ	1881	42694	-	-	-	-	1881.000	1881.001
*β*_*h*_	0	4	2.378	0.802	2.444	0.832	1.717	1.092
*β*_*m*_	0	4	0.104	0.365	0.097	0.326	0.134	0.226
*θ*_*h*_	0.7	1.75	0.969	1.177	0.850	1.110	0.790	0.827
*θ*_*m*_	0.58	0.88	0.580	0.580	0.580	0.580	0.580	0.580
*γ*_*h*_	0.5	1.75	1.750	1.750	1.750	1.749	1.750	1.750
*μ*_*m*_	0.16	0.2	0.196	0.197	0.196	0.198	0.196	0.196

For all of three models, some parameters took the same value in the best estimation such as the transition rate from exposed to infectious mosquitoes, *θ*_*m*_, mortality rate in mosquitoes, *μ*_*m*_, and the recovery rate in humans, *γ*_*h*_. The same behavior was shown by the initial condition of susceptible and infectious mosquitoes, $M_{s_{0}}$ and $M_{i_{0}}$, but also for the initial conditions of exposed humans, $H_{e_{0}}$. In contrast, for models (1) and (2), the fraction of female mosquitoes hatched from all eggs, *f*, and the carrying capacity, *C*, they all took the same value in the best estimation. However, we found that their values were on the edge of their biologically plausible ranges.

Observe that for the per capita oviposition rate, *δ*, and the effective per capita oviposition rate, *ρ*, their values and their ranges were almost the same, since the results of experimental assays with Bello’s vector population showed that the fraction of eggs hatching to larvae, *k*, ranges from 97% to 100%.

**Fig 2:**
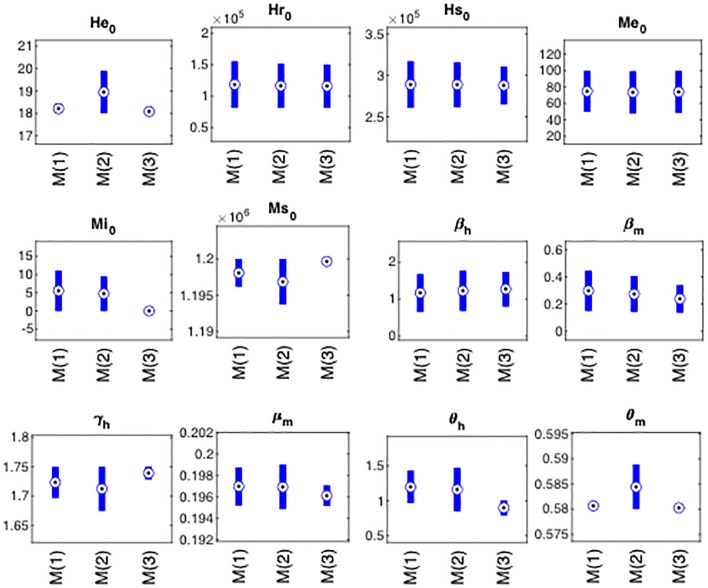
Analysis of parameter intervals after estimation process for models (1)–(3). Here we compare some traditional confidence intervals for the 12 parameters (6 initial conditions and 6 parameters) shared by the three models. To achieve these confidence intervals we remove 5% upper and 5% lower of the results vector for each parameter after estimation process. The boxplots represent the confidence interval of each parameter for every model: M1 is the label for model (1), M2 for model (2), and M3 for model (3). The values estimated for the 12 parameters did not differ significantly from one model to the other, except for the case of *θ*_*h*_ whose interval slightly differs from model (1) to model (3). It is noticeable that the intervals tend to be more punctual for the case of model (3), followed by model (1), while model (2) has the widest of intervals.

Fig 2 shows that the intervals for each parameter range were wider for model (2) than for the other two models. Note that, a few parameters for model (3) converge to a single point value, in contrast with the other models.

### Sensitivity analysis

**Fig 3:**
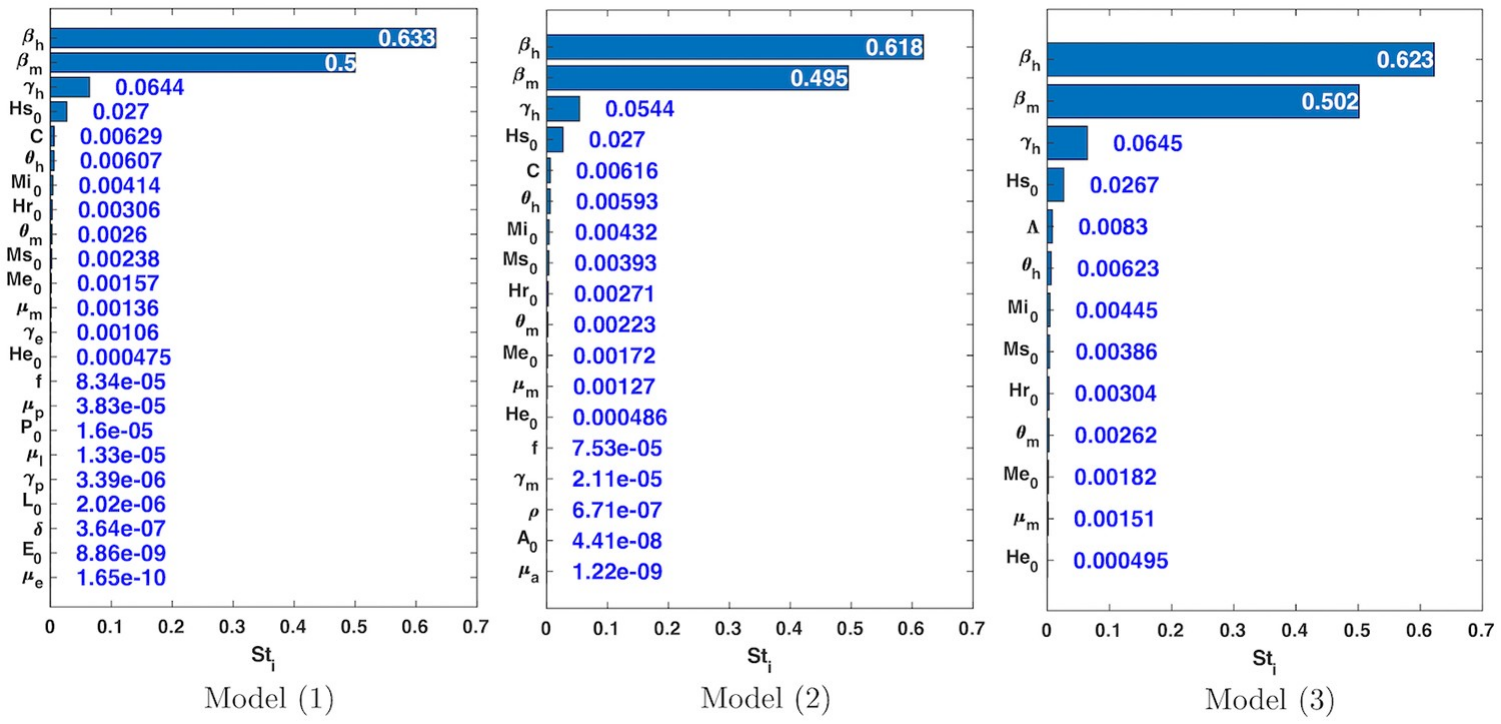
Global sensitivity analysis for models (1)–(3). Here we show the total-order sensitivity indices $\left(S_{t_{i}}\right)$ we obtained from global sensitivity analysis exploring the space of parameters through 10000 samples for each model (up to 125000 simulations for model (1)). We achieved a reliability indicator of 99.7% for model (1), 99.9% for model (2), and 99.8% for model (3). It is noticeable that most of the variance can be linked to three of the parameters for the three models, i.e., those three parameters mostly determine the behavior of the models. Besides, the ranking of relevance of the parameters is almost the same for the models.

**Fig 4:**
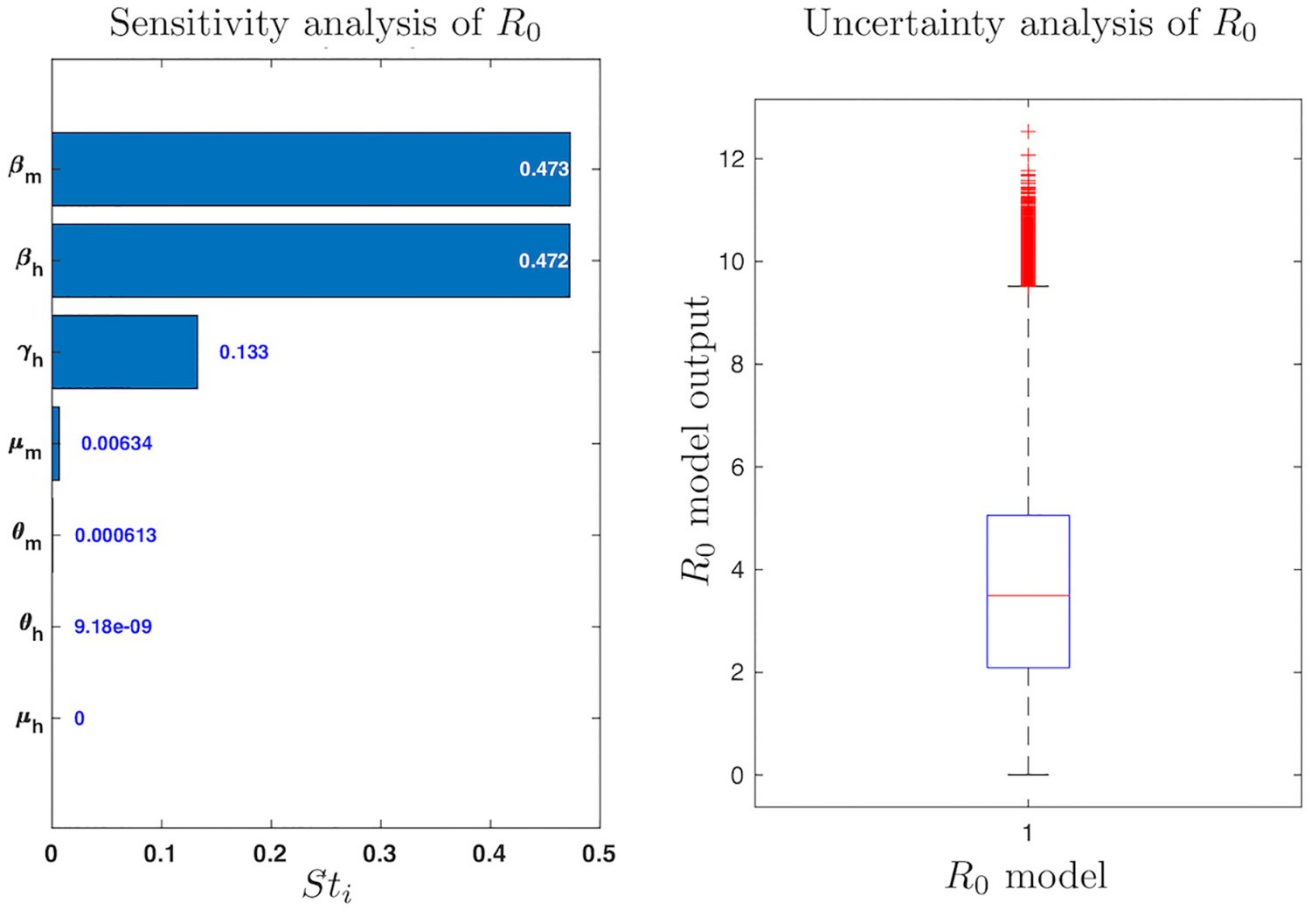
Sensitivity and uncertainty analysis for the basic reproductive number, *R*_0_. We perform UA/SA with 100000 samples (up to 450000 simulations), getting a reliability indicator of 100%. From the SA it is noticeable that the same three parameters that almost determine the behavior of the models (Fig 3) are also the most relevant ones here. On the other hand, the UA shows that the range of the *R*_0_ model is approximately [0 − 12], though most of the outputs are concentrated in low values ([0 − 5]).

We perform global sensitivity analysis for all three models and for the basic reproductive number, *R*_0_. Fig 3 shows that the least sensitive parameters for model (1) and model (2) are those corresponding to the stages of vector development, (eggs, larvae, pupa and aquatic phase) (see Fig 3(a) and 3(b)). While the most sensitive parameters in models (1)–(3) and in the production of secondary infections of dengue in Bello municipality, *R*_0_, were the transmission rate from mosquito to human, *β*_*h*_, the transmission rate from human to mosquito, *β*_*m*_, and the recovery rate, *γ*_*h*_ (Figs 3 and 4(a)). Additionally, Table 4 shows the results of the relevance measure (5) for each model. Before calculating this measure we performed an standardization of the values showed in Fig 3, to make these results comparable.

**Table 4:** Relevance measure for models (1)–(3).

Model	Ψ_1_	Ψ_2_	Ψ_3_	Ψ_4_	Ψ_5_
Model (1)	0.52	0.61	0.78	0.87	0.91
Model (2)	0.67	0.72	0.83	0.83	0.89
Model (3)	0.92	1	1	1	1

The columns of this table are the relevance measure in (5) assuming that $S_{t_{i}}<\Psi_{i}$ implies $S_{t_{i}}=0$, over five levels of Ψ_*i*_: Ψ_1_ = 10^−3^, Ψ_2_ = 10^−4^, Ψ_3_ = 10^−5^, Ψ_4_ = 10^−6^, and Ψ_5_ = 10^−7^, respectively. A model is better than other, if its relevance measure is closer to one, i.e., fewer non-relevant parameters.

On the other hand, since the *R*_0_ model is not a system of ODEs, it is much less expensive to calculate its value and hence, to perform UA/SA. Fig 4(b) shows the behavior of the values of *R*_0_ for 100000 parameter combinations. This result suggest that there is no significant difference between the results obtained from performing global sensitivity analysis and local sensitivity analysis on *R*_0_. The median of the *R*_0_ for these parameter combinations is between two and five. While it is difficult to find combinations of parameters that produce an *R*_0_ greater than ten. These result are consistent with the values of *R*_0_ reported in literature for dengue [47].

### Structural identifiability

For models (1)–(3) we evaluated if these are locally identifiable from the number of cumulative dengue cases reported by official entities only. Also, we fixed the values of human mortality rate, *μ*_*h*_, the initial condition for infectious humans, $H_{i_{0}}$, and the transition rate from larvae to pupae *γ*_*l*_ according to ranges obtained from experimental assays (see Table 1).

Table 5 shows the parameters are not locally identifiable for any of the models. For all models, we obtained that the parameters which describes the development stages of the vector, the recruitment rate in adult population and the initial conditions for vector population were not identifiable from the cumulative number of dengue cases in humans. However, the number of non-identifiable parameters, which should be assumed to be known to obtain a locally structural identifiable system for models (1), (2) and (3) are four, three and one respectively. These numbers correspond to the minimal necessary information that makes the identifiability matrix (Jacobian matrix) have full range.

**Table 5:** Non-structurally identifiable parameters and initial conditions for models (1)–(3).

Model	Non-structural identifiable			
	Parameters		Initial Conditions	
Model (1)	*δ*	Per capita oviposition rate	*E*(0)	Eggs
	*C*	Carrying capacity of the environment	*L*(0)	Larvae
	*γ*_*e*_	Transition rate from eggs to larvae	*P*(0)	Pupae
	*μ*_*e*_	Mortality rate in eggs phase	*M*_*s*_(0)	Susceptible mosquitoes
	*γ*_*p*_	Transition rate from pupae to the adult phase	*M*_*e*_(0)	Exposed mosquitoes
	*μ*_*p*_	Mortality rate in pupae phase	*M*_*i*_(0)	Infectious mosquitoes
	*f*	Fraction of female mosquitoes hatched from all eggs		
Model (2)	*ρ*	Effective per capita oviposition rate	*A*(0)	Aquatic phase
	*C*	Carrying capacity of the environment	*M*_*s*_(0)	Susceptible mosquitoes
	*γ*_*m*_	Transition rate from the aquatic phase to the adult phase	*M*_*e*_(0)	Exposed mosquitoes
	*μ*_*a*_	Mortality rate in the aquatic phase	*M*_*i*_(0)	Infectious mosquitoes
	*f*	Fraction of female mosquitoes hatched from all eggs		
Model (3)	Λ	Recruitment rate	*M*_*e*_(0)	Exposed mosquitoes
			*M*_*s*_(0)	Susceptible mosquitoes
			*M*_*i*_(0)	Infectious mosquitoes

It is possible to obtain locally structural models by using the results from the sensitivity analysis (see Fig 3). For instance, for model (1) we can fix the parameters *δ*, *μ*_*e*_, *γ*_*p*_ and the initial condition for eggs, *E*_0_. For model (2) we can fix the parameters *μ*_*a*_, *ρ*, and the initial condition for the aquatic phase, *A*_0_. For model (3) we can fix the initial condition for exposed mosquitoes, $M_{e_{0}}$.

### Practical identifiability

**Fig 5:**
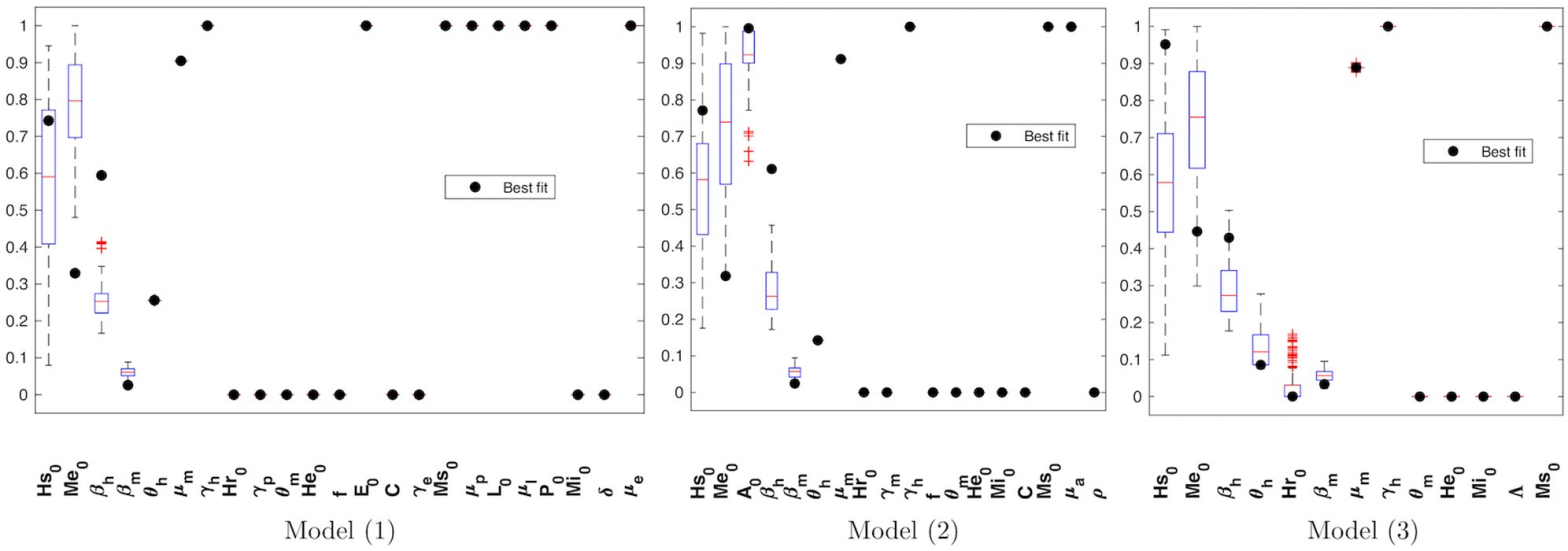
Boxplot identifiability analysis for models (1)–(3). Parameters of each model were ordered from lowest to highest identifiability. The black point represents the value of each parameter for the best estimation (best fit for the dengue real data of Bello municipality). We removed the outliers using the MATLAB function filloutliers and normalized the data with respect to the estimation interval for each parameter before plotting. Almost the same parameters $\left(H_{s_{0}},M_{e_{0}},\beta_{h}\right)$ are unidentifiable for all three models. However, the initial condition of the aquatic stage of the vector (*A*_0_) is not identifiable for model (2), while the transition rate of humans (*θ*_*m*_) and the initial condition of the recovered humans $\left(H_{r_{0}}\right)$ are less identifiable for model (3). It is noticeable that the best estimation for vector-human transition rate (*β*_*h*_) from models (1) and (2) corresponds to an outlier. Also, most of the highly identifiable parameters for the three models are attached to the inferior or superior bound of the estimation interval.

Before performing the practical identifiability analysis through Monte Carlo simulations and correlation matrix, we analyzed the behavior of each parameter in the filtered estimations for each model. We show in Fig 5 a boxplot of the parameter estimations for each model after eliminating their outliers. We normalized the result of each estimation according to the range of each parameter. A parameter is less identifiable when the boxplot is bigger. For all three models, these analyses showed that the initial conditions of susceptible humans and exposed mosquitoes ($H_{s_{0}}$ and $M_{e_{0}}$, respectively) and the transmission rate from mosquito to human, *β*_*h*_, are not identifiable. Moreover, we observed that model (3) has more less identifiable parameters than model (1).

**Fig 6:**
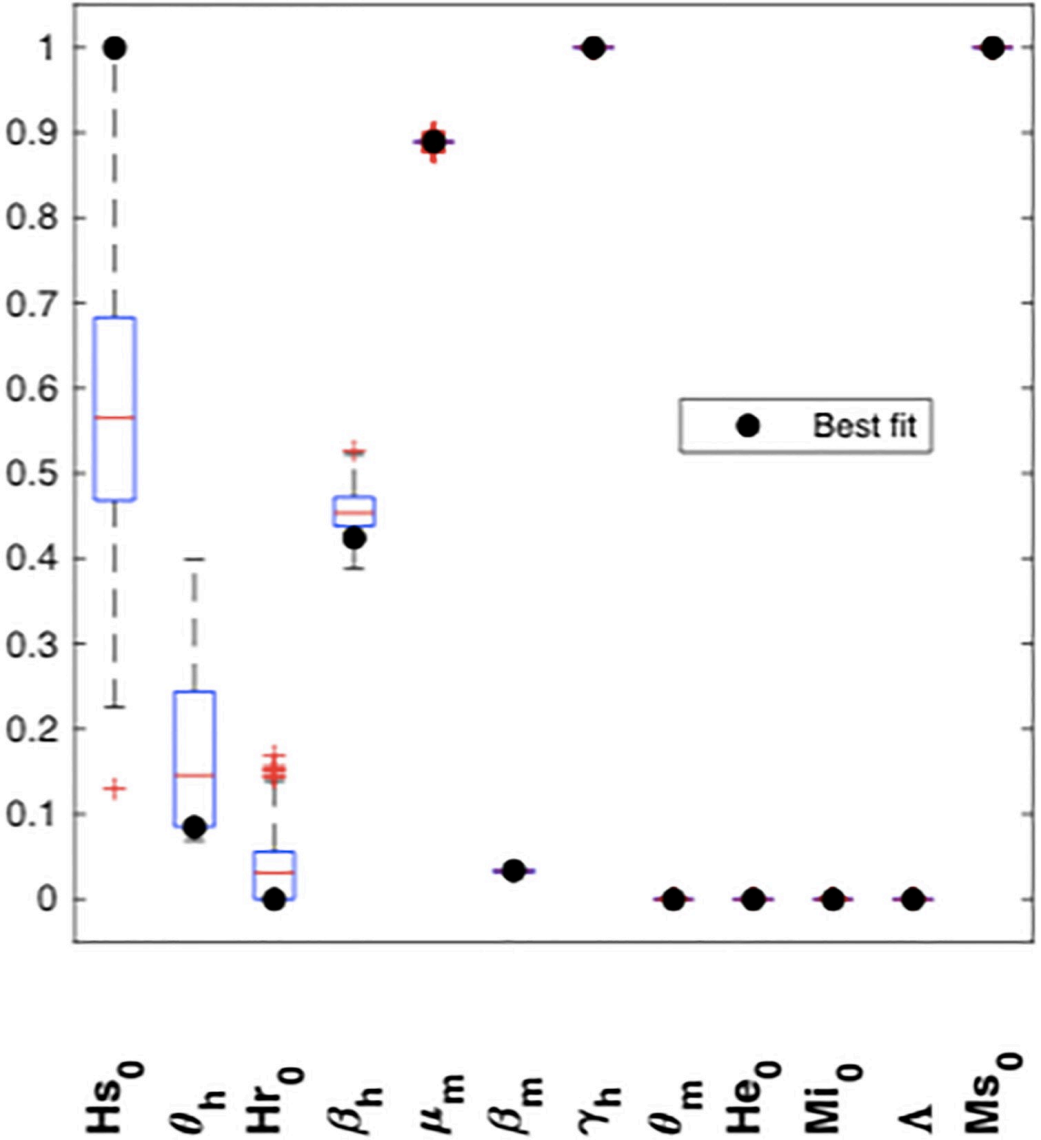
Boxplot of parameters for model (3) after fixing the value $M_{e_{0}}=44.6$. Same as in Fig 5, we remove the outliers and normalized the filtered estimations for model (3). The black dots correspond to the best estimation for the model (best fit to real data, MSE = 642.63). Since the initial condition $M_{e_{0}}$ has been fixed, it has also disappeared from the boxplot. Note that *θ*_*h*_ and $H_{r_{0}}$ are even less identifiable for this model relative to the other models.

According to the results we obtained in the previous section, we fixed the value for the initial condition of exposed mosquitoes, $M_{e_{0}}$ in model (3), to obtain a locally identifiable model. After that, we proceed in the same way as mentioned above, obtaining 257 estimations below the threshold (1% criterion) and *std* = 2.13. Fig 6 shows the results obtained for each parameter. In contrast to the previous result, we observed that the transmission rate from human to mosquito, *β*_*m*_, became more identifiable. On the other hand, the boxplot of *β*_*h*_ decreased, while the boxplots of *θ*_*h*_ and $H_{r_{0}}$ increased, that is, these parameters became less identifiable.

We compared two approaches to determine if models (1)–(3) are practical identifiable. We performed Monte Carlo simulations by generating 1000 random data sets for different measurement error levels and fitting each data set to epidemiological models (1)–(3). Additionally, we consider the non-structural identifiable models, i.e. we estimate all parameters except *μ*_*h*_, *γ*_*l*_ and the initial condition for infectious humans, $H_{i_{0}}$. To determine which parameters are practical identifiable we compute the relative estimation errors (ARE) for each parameter of each model.

We see from Tables 6–8 that the number of identifiable parameters remains constant when the noise in the data increases. For all models we obtained that the AREs for the initial condition of infectious mosquitoes, $M_{i_{0}}$, were high showing that this parameter is sensitive to the noise in the data. Besides, for models (1) and (2) the oviposition rate (*δ*), and the effective oviposition rate (*ρ*), were not practical identifiable parameters, according to the results we obtained from structural identifiability analysis.

**Table 6:** Practical identifiability analysis for each parameter and initial condition of model (1) by Monte Carlo simulations.

Parameter	Estimated value	Error level *σ*_0_%					
		0%	1%	5%	10%	20%	40%
*E*_0_	95000	0	0.1	0.4	0.6	2	5.6
*L*_0_	95000	0	0	0.1	0.3	1.1	2.5
*P*_0_	95000	0	0	0.1	0.3	0.6	1.2
$M_{s_{0}}$	1200000	0	0	0.1	0.3	0.5	1.0
$M_{e_{0}}$	32.938	0	0.1	0.5	1.1	2.6	5.9
$M_{i_{0}}$	0	0	1.37 × 10^9^	9.77 × 10^9^	2.27 × 10^10^	5.34 × 10^10^	1.46 × 10^11^
$H_{s_{0}}$	301842.132	0	0	0.1	0.1	0.3	1.3
$H_{e_{0}}$	18	0	0	0.4	1.1	2.5	7.8
$H_{r_{0}}$	81405.0	0	0.2	0.3	0.4	0.7	1.7
*δ*	20	0	1.5	16.8	36	81.6	217.5
*γ*_*e*_	1.4	0	0	0.1	0.3	0.8	2
*γ*_*p*_	2.33	0	0	0.1	0.2	0.3	0.7
*μ*_*e*_	0.11	0	0.1	1.5	3.3	7.5	19.8
*μ*_*l*_	0.18	0	0	0.2	0.3	0.5	1.4
*μ*_*p*_	0.58	0	0	0.2	0.3	0.6	1.1
*C*	6400	0	0	0.6	1.7	4.2	10.6
*f*	0.35	0	0	0.1	0.2	0.4	0.8
*β*_*h*_	2.378	0	0.1	0.4	0.8	1.7	3.7
*β*_*m*_	0.104	0	0.3	1.2	2.5	5	11.3
*θ*_*h*_	0.969	0	0.2	1.2	2.8	6.9	17.2
*θ*_*m*_	0.58	0	0	0.3	0.5	1	2.2
*γ*_*h*_	1.75	0	0.2	0.3	0.5	0.9	1.9
*μ*_*m*_	0.196	0	0.1	0.4	0.9	1.7	3

ARE for each parameter of model (1) when fitted to cumulative number of reported dengue cases in Bello municipality. The values that are above the error level are shown in gray.

**Table 7:** Practical identifiability analysis for each parameter and initial condition of model (2) by Monte Carlo simulations.

Parameter	Estimated value	Error level *σ*_0_%					
		0%	1%	5%	10%	20%	40%
*A*_0_	17216.859	0	0.5	1	1.9	2.7	7.3
$M_{s_{0}}$	1200000	0	0	0.2	0.3	0.6	1.0
$M_{e_{0}}$	31.839	0	0.1	0.5	1.2	2.8	6.4
$M_{i_{0}}$	0	0	4.51 × 10^13^	2.35 × 10^14^	5.88 × 10^14^	1.58 × 10^15^	3.89 × 10^15^
$H_{s_{0}}$	304026.086	0	0	0.1	0.1	0.3	1.2
$H_{e_{0}}$	18	0	0.1	0.5	1.2	2.9	8.0
$H_{r_{0}}$	81407.485	0	0.2	0.3	0.5	0.9	2.2
*C*	6400	0	0.1	0.9	2.3	5.1	14.1
*f*	0.35	0	0	0.1	0.2	0.5	1.0
*ρ*	19	0	2.9	19.8	38.6	84.2	212.9
*γ*_*m*_	0.875	0	0	0	0.1	0.1	0.3
*μ*_*a*_	0.16	0	0.2	1.5	3.0	6.5	16.4
*β*_*h*_	2.444	0	0.1	0.3	0.8	1.8	3.8
*β*_*m*_	0.097	0	0.3	1.3	2.4	5.3	12.2
*θ*_*h*_	0.85	0	0.3	1.3	2.8	7.6	18.9
*θ*_*m*_	0.58	0	0	0.2	0.5	1.1	2.3
*γ*_*h*_	1.75	0	0.2	0.3	0.5	0.9	2.1
*μ*_*m*_	0.196	0	0.1	0.5	0.9	1.7	3.3

ARE for each parameter of model (2) when fitted to weekly reported dengue cases in Bello municipality. The values that are above the error level are shown in gray.

**Table 8:** Practical identifiability analysis for each parameter and initial condition of model (3) by Monte Carlo simulations.

Parameter	Estimated value	Error level *σ*_0_%					
		0%	1%	5%	10%	20%	40%
$M_{s_{0}}$	1200000	0	0	0.2	0.2	0.4	0.8
$M_{e_{0}}$	44.6	0	0.1	0.7	1.7	3.6	7.7
$M_{i_{0}}$	0	0	3.54 × 10^13^	3.15 × 10^14^	5.82 × 10^14^	1.16 × 10^15^	3.85 × 10^15^
$H_{s_{0}}$	318001.356	0	0	0.1	0.2	0.7	2.1
$H_{e_{0}}$	18	0	0.1	0.6	1.4	3.2	7.8
$H_{r_{0}}$	81439.356	0	0.2	0.4	0.6	1.2	2.0
Λ	1881	0	0.2	1.6	3.9	9.1	22.7
*β*_*h*_	1.717	0	0.1	0.5	1.3	3	5.7
*β*_*m*_	0.134	0	0.3	1.4	3.2	7.7	15.2
*θ*_*h*_	0.79	0	0.3	1.8	4.6	14.1	30.8
*θ*_*m*_	0.58	0	0.1	0.3	0.6	0.8	1.6
*γ*_*h*_	1.75	0	0.2	0.3	0.4	0.5	0.9
*μ*_*m*_	0.196	0	0.1	0.6	1	1.9	3.4

ARE for each parameter of model (3) when fitted to weekly reported dengue cases in Bello municipality. The values that are above the error level are shown in gray.

We analyzed the behavior of the parameters in model (3) after fixing $M_{e_{0}}$. In Table 9 we observe that the relative errors of all parameters except for the initial condition of infectious mosquitoes, $M_{i_{0}}$, are always smaller than the implemented noise. We conclude that even when we consider the locally structural identifiable model (3), $M_{i_{0}}$ remains unidentifiable.

**Table 9:** Practical identifiability analysis for each parameter and initial condition of model (3) by Monte Carlo simulations after we fixed the initial condition of exposed mosquitoes.

Parameter	Estimated value	Error level *σ*_0_%					
		0%	1%	5%	10%	20%	40%
$M_{s_{0}}$	1200000	0	0	0.2	0.3	0.4	0.9
$M_{i_{0}}$	0	0	8.91 × 10^9^	6.27 × 10^10^	1.38 × 10^11^	2.81 × 10^11^	6.26 × 10^11^
$H_{s_{0}}$	321700.801	0	0	0.1	0.3	1.2	2.8
$H_{e_{0}}$	18	0	0.1	0.6	1.6	3.8	7.8
$H_{r_{0}}$	81405.053	0	0.1	0.2	0.4	0.8	1.4
Λ	1881	0	0.1	1.2	3.1	8.8	21.3
*β*_*h*_	1.697	0	0.1	0.6	2.1	5.1	8.7
*β*_*m*_	0.135	0	0.3	1.2	3.9	10.5	19.1
*θ*_*h*_	0.789	0	0.2	1.4	4.8	16.8	31.3
*θ*_*m*_	0.58	0	0	0.2	0.6	0.9	1.6
*γ*_*h*_	1.75	0	0.1	0.3	0.5	0.7	1.1
*μ*_*m*_	0.196	0	0.1	0.5	0.9	1.8	3

ARE for each parameter of model (3) when fitted to weekly reported dengue cases in Bello municipality after we fixed the initial condition of exposed mosquitoes ($M_{e_{0}}=44.6$) to obtain a structural identifiable model. The values that are above the error level are shown in gray.

**Fig 7:**
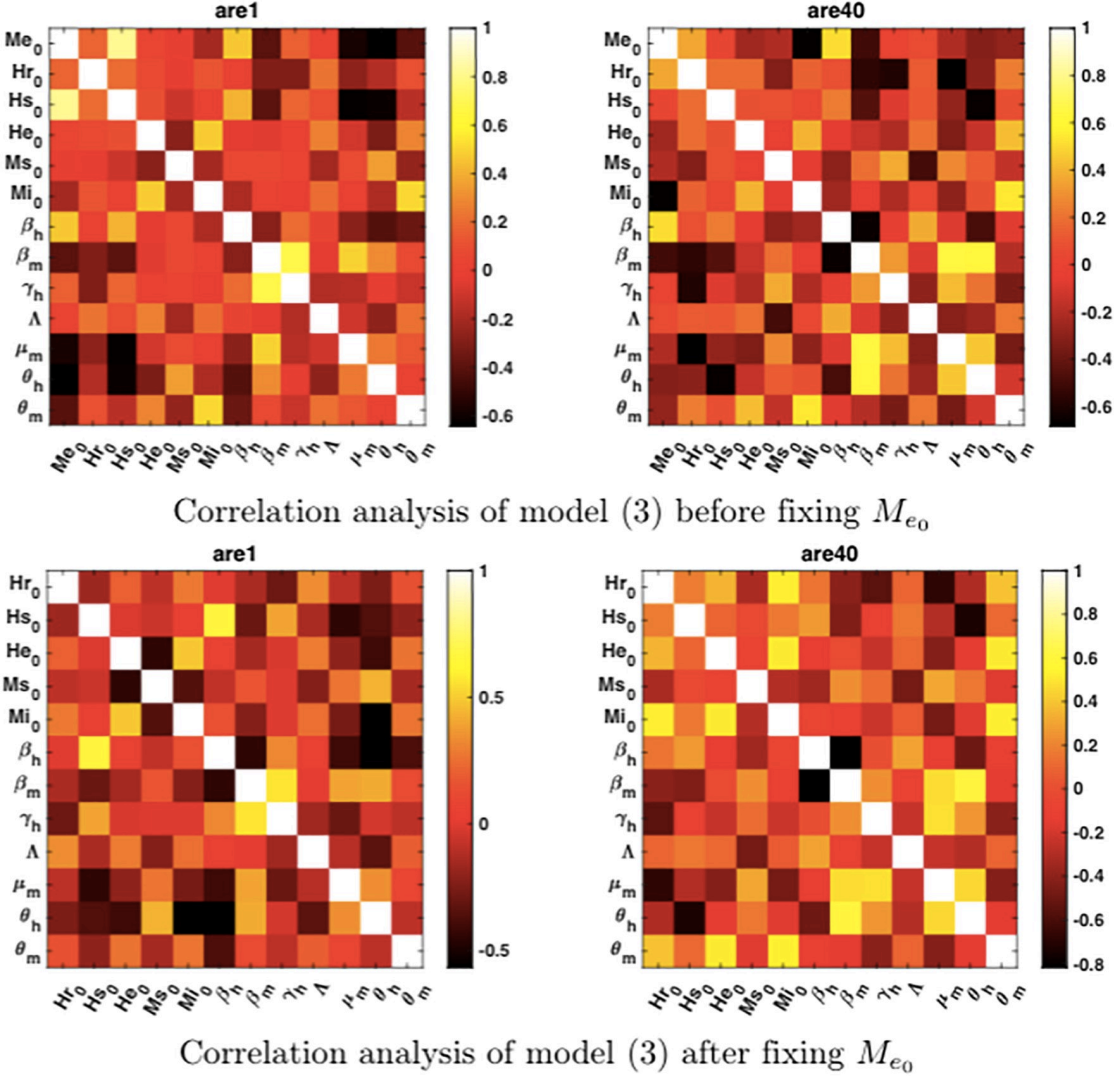
Correlation analysis for model (3) before and after being locally structural identifiable. Correlation matrices for model (3) using the Pearson correlation index. Strong correlation (|*index*| > 0.8) suggest that the parameters are unidentifiable. Here we present the correlation matrices for two noise levels, *σ* = 1% (ARE1) and *σ* = 40% (ARE40). Before we fixed $M_{e_{0}}$ we found a strong positive correlation between $M_{e_{0}}$ and $H_{s_{0}}$ for low noise level; on the other hand, after fixing $M_{e_{0}}$ we found strong negative correlation between transmission rates (*β*_*h*_ and *β*_*m*_), as well as between $H_{s_{0}}$ and *θ*_*h*_ for high noise level. Those negative correlated parameters correspond to the unidentifiable ones from Fig 6.

In addition, we compute the correlation matrix for model (3) before and after we fixed $M_{e_{0}}$, as it can be seen in Fig 7. We found that only few parameters show a strong correlation through the four scenarios we presented. Also, the most noticeable correlations for a given error level were not the same as in the other level. For instance, the transmission rates *β* achieved the highest correlation for an error level of 40%, while $M_{e_{0}}$, $H_{s_{0}}$ and *θ*_*h*_ show the highest correlation for an error level of 1%.

## Discussion

This study set out with the aim of assessing the importance of carrying out sensitivity, uncertainty, and identifiability (structural and practical) analyses to dengue transmission models to determine if all models explain the transmission in the same way or if there are differences that help the researcher choose one model over the others. As far as we know, this is the first study in which the performance of different models is evaluated through the aforementioned analyses to determine which model is more reliable to simulate and identify the main parameters involved in the transmission of dengue, taking into account the available information to fit the model and to define the parameter intervals.

Numerous studies have attempted to explain the transmission of dengue disease, taking into account several considerations such as a variable human population [6], effects of vector control on dengue transmission [25], the existence of multiple serotypes [8], the effect of temperature in the transmission and development of the vector [48–50], among others. A more detailed review is provided in [9, 10]. However, despite the good fitting results obtained with these models and the biological explanation that supports the incorporation of these factors, few studies have evaluated the relation between the model formulation and the available data to obtain more reliable and accurate estimations. For instance, in [12, 51] the authors evaluated the performance and reliability of the fitting data of different models only taking into account the results of the structural and practical identifiability analyses. While others studies only perform sensitivity analyses on the basic reproductive number, *R*_0_, to determine which parameters are more important in the production of secondary cases [52, 53]. However, we found that it is necessary to carry out these analyses jointly because they are complementary.

We show that models (1)–(3) can be fitted to the cumulative number of reported dengue cases with almost the same cost function (Fig 1). However, models of greater complexity (more parameters and more state variables) require more information to guarantee that the model is locally structural identifiable, and in this way, also guarantee that the parameter estimation problem can be solved in a unique way, assuming noise-free data and error-free model (see Table 5). Additionally, since all the parameters that interact with the human population directly are locally identifiable from the cumulative number of reported dengue cases, the structural identifiability analysis suggests that in order to have structural identifiable models, it is necessary to collect information about the transmission dynamics of the vector.

Another important finding was that the least sensitive parameters in the models were those that describe the development stages of the vector (see Fig 3). This is an indicator that shows it is unnecessary to consider more state variables when we want to explain an epidemic outbreak, since these equations do not provide new information, and do not help to identify other parameters. Conversely, those state variables provide more degrees of freedom that can spread more errors during the estimation process, also increasing the required computing time to achieve similar results to those achieved by less complex models. Perhaps, the most significant finding is the application of the relevance measure (5) to each model, which is a clear indicator for the choice of one model over the others (see Table 4). We strongly believe that these results were reliable since the reliability indicator was very close to 100% for each model (see Fig 3).

Notwithstanding, as we see from Tables 6–9, the practical identifiability analysis through error levels does not provide interesting information about the identifiability of the models, which turns out more evident as we contrast those results with the ones we presented in Fig 5. For instance, results from error levels approach suggest that the unidentifiable parameters are the initial condition of infectious mosquitoes ($M_{i_{0}}$) and the oviposition rate (*δ* or *ρ*). However, the analysis through the boxplots of filtered estimations points out that the main unidentifiable parameters are $H_{s_{0}}$ and $M_{e_{0}}$, followed by the transmission rates (*β*_*m*_ and *β*_*h*_) and few more parameters for the case of models (2) and (3). We think that those contradictory results from both approximations could be attributed, to some extent, to the fact that error levels approach is a local method instead of a global one, as the boxplot approach. Bearing in mind the nature of the methods, we can argue that the error levels approach constitutes an exploration of the behavior of a given minimum. Thus, the method itself does not reveal the presence of identifiability issues due to local or even global minima. On the other hand, the boxplot approach attempts to explore the whole space of parameters, and after the filtering process, it even yields information about the convergence of the parameters to the global minimum. Hence, assuming that filtered estimations share an equally good cost function (i.e., that we can not differentiate among their fit) all the parameter distributions must tend either to be punctual or to have multiple global minima. We also think that the advantages we exposed about the boxplot approach are supported by the results from the correlation matrix plots in Fig 7. Even though the criterion for strong interactions was seldom overcome, it is noticeable that those rows (or columns) that correspond to the less identifiable parameters for model (3) are also the rows that present the strongest interactions for each matrix (darker or brighter colors).

A final aspect that should be mentioned is that for all models, (1)–(3), we found that the basic reproductive number is given by the same expression (15) through the application of *Next Generation Matrix* operator [30]. This result is coherent since the infected subsystem is the same for all three models (10). Additionally, the sensitivity and uncertainty analysis of *R*_0_ suggest that different results of parameter estimation produce different model fit to real data, while different values of parameter estimation can produce the same value of *R*_0_ (Fig 4).

In summary, Section Results allow us to conclude that if we are only interested in formulating a dengue transmission model capable of replicating the occurrence of the outbreak as the aforementioned Bello case, taking into account that the only information available is the number of new cases per week, the most appropriate model is model (3). It is important to notice that the election of this model does not depend only on the complexity of the model, since model (2) had worst performance than model (1) in analyses as the relevance measure, and the practical identifiability analysis through the boxplots.

Further research might explore the behavior of these analyses on models that consider control strategies at all stages of vector development, models that include several virus serotypes, and models that consider the change in the parameters of the model when the temperature, relative humidity, and precipitation change. It is important to carry out these analyses when information about these hypotheses is available to determine which model is more appropriate. Moreover, it is important to examine more closely the links between the parameters estimated, the output of the other states of the model, and the expression for the basic reproductive number, *R*_0_ to provide more biological meaning in model results. Hence, one of the most remarkable features in the trajectory of the model states through the simulation of the epidemic outbreak turned up as we focused on those states that simulate the vector population. It is noticeable from S2 Fig that, for all three models, the initial condition of the vector states (except for $M_{i_{0}}$) was much higher than those values the states reach at the end of the simulation, i.e., said states were starting from a point far from the stationary state. The behavior of the vector population gains more relevance as we take into account the results from Fig 5, that presents the distribution of the estimated parameters after the filtering process; as it can be seen, all the parameters linked to large initial non-infective vector population are attached to the superior bound of their intervals, while the parameters linked to the vector proliferation (vector population renewal) are attached to the inferior bound of their intervals.

Additionally, the different death rates (*μ*) are attached to high values of the intervals or directly to the superior bounds also. We think that it is possible to explain the phenomenon we described above as an initial (not-considered) perturbation of the system, and hence, we decided to call it the *initial pulse effect*. Since we did not consider variables to handle or incorporate such kind of phenomena to the model, we assume that it was numerically taken into account by the solver during estimation process by setting the initial vector population large as possible, and then allowing a rapid transition to a stationary state determined by the lowest values for the survival of immature vector states, the carrying capacity, and the oviposition rate. In this way, the epidemic outbreak occurs as a secondary process linked to the transition of the vector population to its stationary state. Our belief in this statement is based on the *R*_0_ values from the filtered estimations. As can be seen in S3 Fig, most of the *R*_0_ values for the estimations fell within the range of [0.5 − 0.6] (none of the 1000 estimations achieved an *R*_0_ greater than one), which suggest, from a general interpretation of *R*_0_, that there was no epidemic outbreak in Bello municipality. However, it is necessary to point out that one of the main required assumptions for calculating *R*_0_ through the next generation matrix approach is that both of the vector and human populations are at equilibrium state, which clearly does not hold for the scenarios we estimated. Hence, the *initial pulse effect* can be summarized as follows: the susceptible vector population is as high as possible considering its equilibrium at the start of the epidemic outbreak, then, as the term $\frac{M_{s}^{*}}{M_{s}}$ from Eq (14) suggests, the disease starts spreading in the human population; however, as we step forward in the simulation, the susceptible vector population tends to equilibrium where its respective *R*_0_ is lower than one (as we calculated it), firstly slowing down the spread rate of the outbreak until the disease begins disappearing from the human population.

The *initial pulse effect* points out that the next generation matrix approach could not be appropriate for the *R*_0_ calculation of vector-borne diseases whose vector population is far from equilibrium at the start of the simulation. Furthermore, if it is possible to trigger an outbreak just introducing some perturbations over the vector dynamics such that the vector population is taken away from its equilibrium state, then it should be possible to simulate several epidemic outbreaks and even the endemic behavior through consecutive perturbations. Finally, although we argue that the calculation of *R*_0_ through next-generation matrix approach does not seems like an appropriate strategy, it is remarkable that the *R*_0_ interpretation from [54], which states that *R*_0_ > 1 implies the establishment of the disease in the population while *R*_0_ < 1 implies the disappearing of the disease, actually holds.

## Supporting information

S1 Fig Flowgraphs for models (1)–(3). Diagrams summarizing the transitions from one compartment to another for each model.
(PDF)

S2 Fig Behavior of all states variables for model (1). (PDF)

S3 Fig *R*_0_ values for the best estimations of each model. (PDF)

S1 File An elementary mathematical framework for variance-based sensitivity analysis: Sensitivity indices and their meaning. (PDF)
