# Supplementary material for: Sensitivity, uncertainty and identifiability analyses to define a dengue transmission model with real data of an endemic municipality of Colombia
Source: PLoS One. 2020 Mar 11;15(3):e0229668. doi: 10.1371/journal.pone.0229668 (PMC7065780; doi:10.1371/journal.pone.0229668)
Supplement: S1 Fig — Diagrams summarizing the transitions from one compartment to another for each model. (PDF) [file pone.0229668.s001.pdf]

Supporting information

S1 Fig. Flowgraphs for models (1)–(3)**.** Diagrams summarizing the transitions from one compartment to another for each model.

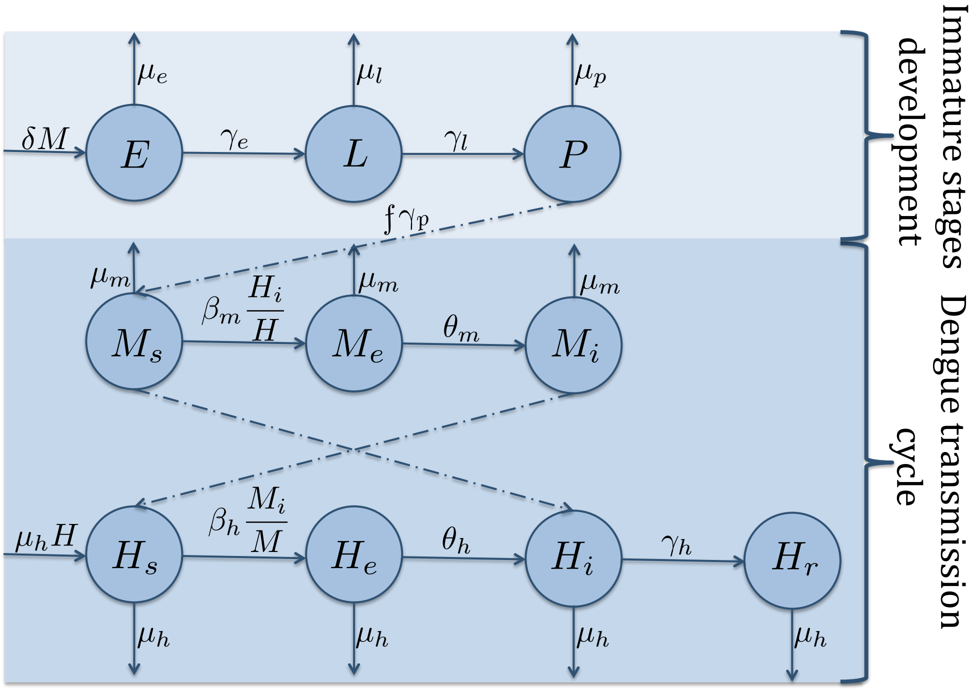

Fig 1. Flow-graph of model (1)**.** In this model all stages of vector were included. The diagram summarizing the transitions from one compartment to another. It is assumed that the mosquito population is divided into immature stages (mosquito) and adult females (mosquito).

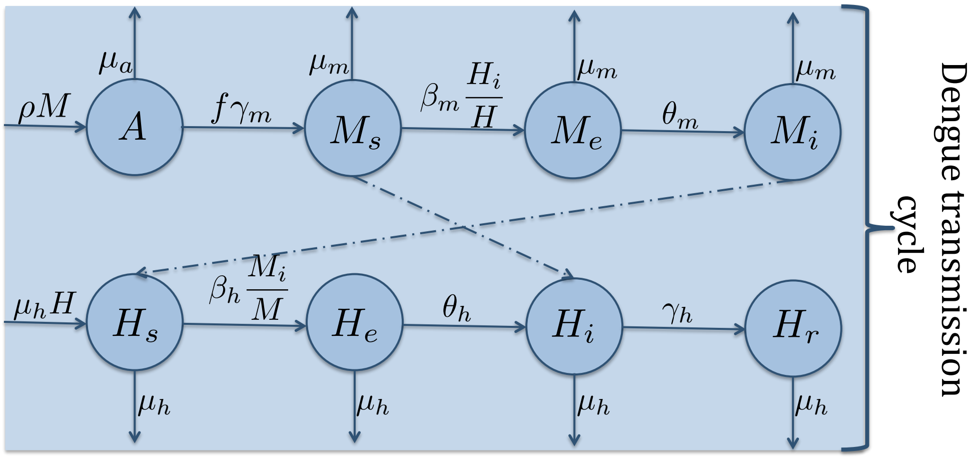

Fig 2. Flow-graph of model (2)**.** Flow diagram summarizing the transitions from one compartment to another for model (2) is shown. In this model, the aquatic phase (larvae and pupae) was grouped in the variable A. It is assumed that the mosquito population is divided into aquatic phase A and adult females M.

Flow-graph of model (3)**.**

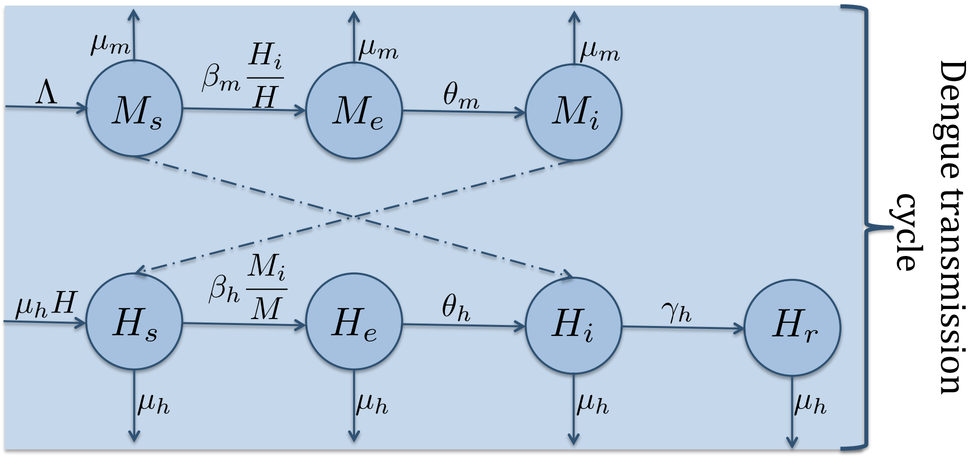

Fig 3. Dengue transmission model. Flow diagram summarizing the transitions from one compartment to another for model (3) is shown. It is assumed that the mosquito population is only adult females M.
