# Supplementary material for: Sensitivity, uncertainty and identifiability analyses to define a dengue transmission model with real data of an endemic municipality of Colombia
Source: PLoS One. 2020 Mar 11;15(3):e0229668. doi: 10.1371/journal.pone.0229668 (PMC7065780; doi:10.1371/journal.pone.0229668)
Supplement: S2 Fig — (PDF) [file pone.0229668.s002.pdf]

Supporting information

S2 Fig. Behavior of all states variables for model (1)**.**

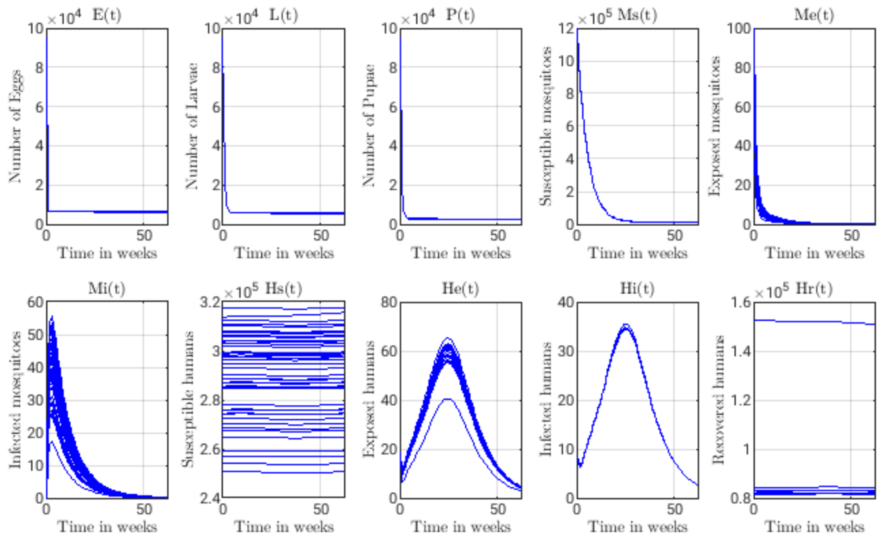

Fig 1. All states from filtered estimations in model (1)**.** As it can be seen, all model states linked to the vector population and its developmental stages start from a value much higher than their equilibrium value. Thus, all the epidemic outbreak occurs during the transition to equilibrium of the vector population. We noticed that the same behavior is presented for models (2) and (3).
