# Supplementary material for: Sensitivity, uncertainty and identifiability analyses to define a dengue transmission model with real data of an endemic municipality of Colombia
Source: PLoS One. 2020 Mar 11;15(3):e0229668. doi: 10.1371/journal.pone.0229668 (PMC7065780; doi:10.1371/journal.pone.0229668)
Supplement: S3 Fig — (PDF) [file pone.0229668.s003.pdf]

Supporting information

S3 Fig. R_0 values for the best estimations of each model.

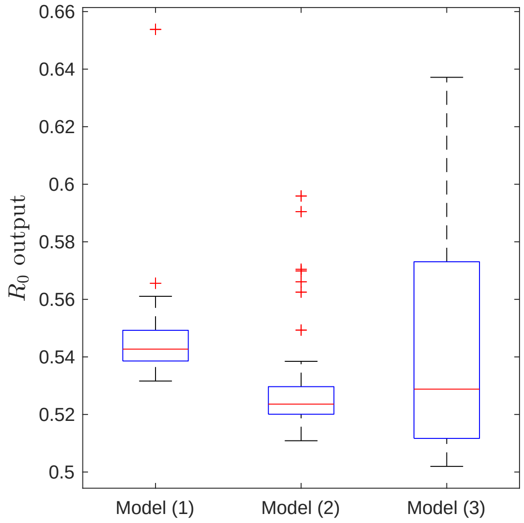

Fig 1. R_0 value for the filtered estimations for models (1)–(3). We calculate the corresponding R_0 value for the 136 estimations from model (1), 158 estimations from model (2), and 476 estimations from model (3). As it can be seen in the boxplot, none of the estimated parameters correspond to a R_0 value greater than 1, indeed, most of the estimations correspond to R_0 values among 0.5 and 0.6, being model (3) the one that shows the major variability for R_0.
