# Supplementary material for: Sensitivity, uncertainty and identifiability analyses to define a dengue transmission model with real data of an endemic municipality of Colombia
Source: PLoS One. 2020 Mar 11;15(3):e0229668. doi: 10.1371/journal.pone.0229668 (PMC7065780; doi:10.1371/journal.pone.0229668)
Supplement: S1 File — (PDF) [file pone.0229668.s004.pdf]

Supporting information

S4 File: An elementary mathematical framework for variance-based sensitivity analysis (Sensitivity indices and their meaning).

Following [1] [2] and [3], the variance-based framework is given as follows:

Consider a model ϕ with deterministic time-dependent (t) model function f of the form $\phi(X) = f(X,t)$ and k input factors $X = [X_1, X_2, \ldots, X_k]$ (the term factor group both of initial state conditions and model parameters) with its respective interval. Defining X_i as the $i-$th factor, then $X_i \in \left[\underline{I_i}, \overline{I_i}\right] = I_i$, where I_i is the factor interval and $\underline{I_i}, \overline{I_i}$ represent their lower and upper bounds, respectively. Hence, the space of factors of the model is defined as $\Omega = \{I_1\} \times \cdots \times \{I_k\}$.

Being ϕ an integrable function defined in Ω, we can obtain an equivalent high-dimensional model representation with 2^k terms (1) that exists and is unique if the factors are independent [2] [3]. From now on, $X_{\sim i}$ denotes the vector of all factors but X_i, $V_{X_i}(\cdot)$ and $E_{X_i}(\cdot)$ denote the variance and mean of argument $(\cdot)$ taken over X_i, respectively; and $V_{X_{\sim i}}(\cdot)$ and $E_{X_{\sim i}}(\cdot)$ denote the variance and mean of argument $(\cdot)$ taken over all factors but X_i, respectively.

$$\phi(X) = \phi_0 + \sum_{i=1}^{k} \phi_i(X_i) + \sum_{1 \le i \le j \le k} \phi_{i,j}(X_i, X_j) + \ldots + \phi_{1,2,\ldots,k}(X_1, X_2, \ldots, X_k) \tag{1}$$

where

$$\begin{aligned} \phi_0 &= E(\phi(X)) \\ \phi_i &= E_{X_{\sim i}}(\phi(X)|X_i) - \phi_0 \\ \phi_{ij} &= E_{X_{\sim ij}}(\phi(X)|X_i, X_j) - \phi_i - \phi_j - \phi_0 \end{aligned} \tag{2}$$

and so on for higher orders. Also, it is possible to state a relation between functions $\phi_{i1,i2,\ldots,ik}$ and partial variances:

$$\begin{aligned} V_i &= V(\phi_i(X_i)) = V_{X_i}[E_{X \sim i}(\phi(X)|X_i)] \\ V_{i,j} &= V(\phi_{i,j}(X_i, X_j)) \\ &= V_{X_i,X_j}[E_{X_{\sim i,j}}(\phi(X)|X_i, X_j)] - V_{X_i}[E_{X \sim i}(\phi(X)|X_i)] - V_{X_j}[E_{X \sim j}(\phi(X)|X_j)] \end{aligned} \tag{3}$$

and so on for higher orders. All terms are linked by:

$$V(\phi(X)) = \sum_{i=1}^{k} V_i + \sum_{1 \le i \le j \le k} V_{i,j} + \ldots + V_{1,2,\ldots,k} \tag{4}$$

Thus, dividing both sides of (4) by $V(\phi(X))$, we obtain:

$$1 = \sum_{i=1}^{k} \frac{V_i}{V(\phi(X))} + \sum_{1 \le i \le j \le k} \frac{V_{i,j}}{V(\phi(X))} + \ldots + \frac{V_{1,2,\ldots,k}}{V(\phi(X))} \tag{5}$$

Renaming $S_i = \frac{V_i}{V(\phi(X))}, S_{i,j} = \frac{V_{i,j}}{V(\phi(X))}, \ldots, S_{1,2,\ldots,k} = \frac{V_{1,2,\ldots,k}}{V(\phi(X))}$. Then we call S_i as the first order sensitivity index of the $i-$th factor, $S_{i,j}$ as the second order sensitivity index of the $i-$th and $j-$th factors, and so on. First order indices measure the

normalized expected contribution to model output variance from $i-$th factor alone, second order indices measure the normalized expected contribution to model output variance from the interaction between $i-$th and $j-$th factor, and so on. Further, as shown in [3], due to the identity

$$V_{X_i}[E_{X_{\sim i}}(\phi(X)|X_i)] + E_{X_i}[V_{X_{\sim i}}(\phi(X)|X_i)] = V(\phi(X)) \tag{6}$$

it is also possible to quantify the whole contribution of each factor (its single contribution plus second order contributions, plus higher order contributions) to the model output variance as:

$$S_{Ti} = \frac{E_{X_{\sim i}}[V_{X_i}(\phi(X)|X_{\sim i})]}{V(\phi(X))} = 1 - \frac{V_{X_{\sim i}}[E_{X_i}(\phi(X)|X_{\sim i})]}{V(\phi(X))} \tag{7}$$

which makes sense since $V_{X_{\sim i}}[E_{X_i}(\phi(X)|X_{\sim i})]$ is the first order effect of $X_{\sim i}$, hence, $V(\phi(X)) - V_{X_{\sim i}}[E_{X_i}(\phi(X)|X_{\sim i})]$ is the contribution of all terms in the variance decomposition which do include the $i-$th factor. There are several works in the literature that propose estimators and their respective algorithms for the calculation of S_i and S_{Ti} (see for instance [3] [4]). In these papers, for sensitivity indices calculation we applied a routine implemented in GSUA-CSB toolbox [5] based on the work in [4], which is specially recommended to estimate sensitivity indices when the model output is time-dependent. Finally, it is worth to state the following inequality from [6]:

$$\sum_{i=1}^{k} S_i \leq 1 \leq \sum_{i=1}^{k} S_{Ti} \tag{8}$$

Equality holds when the model is purely additive, i.e., there are no interactions among factors. We say a model has no strong interactions when $\sum_{i=1}^{k} S_i > 0.7$, in which case we conclude that local approaches for sensitivity analysis are by far good enough to study error propagation in the model (uncertainty and sensitivity).

References

1. Sobol IM. Global sensitivity indices for nonlinear mathematical models and their Monte Carlo estimates. Mathematics and computers in simulation. 2001;55:271–280.

2. Chastaing G, Gamboa F, Prieur C. Generalized Hoeffding-Sobol decomposition for dependent variables - application to sensitivity analysis. Electronic Journal of Statistics. 2012;6(0):2420–2448. doi:10.1214/12-ejs749.

3. Saltelli A, Annoni P, Azzini I, Campolongo F, Ratto M, Tarantola S. Variance based sensitivity analysis of model output. Design and estimator for the total sensitivity index. Computer physics communications. 2010;181:259–270.

4. Xiao S, Lu Z, Wang P. Multivariate Global Sensitivity Analysis Based on Distance Components Decomposition. Risk Analysis. 2018;38(12):2703–2721. doi:10.1111/risa.13133.

5. Rojas-Díaz, Daniel and Vélez-Sánchez, Carlos Mario. drojasd/GSUA-CSB: GSUA-CSB v1.0; 2019. Available from: `https://zenodo.org/record/3383316`.

6. Saltelli A, Ratto M, Andres T, Campolongo F, Cariboni J, Gatelli D, et al. Global sensitivity analysis: The primer. Chichester, England: John Wiley & Sons; 2008.
